# Conformational Stability of Fibrillar Amyloid-Beta Oligomers via Protofilament Pair Formation – A Systematic Computational Study

**DOI:** 10.1371/journal.pone.0070521

**Published:** 2013-07-31

**Authors:** Anna Kahler, Heinrich Sticht, Anselm H. C. Horn

**Affiliations:** Bioinformatik, Institut für Biochemie, Friedrich-Alexander-Universität Erlangen-Nürnberg, Erlangen, Germany; German Research School for Simulation Science, Germany

## Abstract

Amyloid-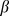 (A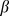) oligomers play a crucial role in Alzheimer’s disease due to their neurotoxic aggregation properties. Fibrillar A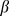 oligomerization can lead to protofilaments and protofilament pairs via oligomer elongation and oligomer association, respectively. Small fibrillar oligomers adopt the protofilament topology, whereas fibrils contain at least protofilament pairs. To date, the underlying growth mechanism from oligomers to the mature fibril still remains to be elucidated. Here, we performed all-atom molecular dynamics simulations in explicit solvent on single layer-like protofilaments and fibril-like protofilament pairs of different size ranging from the tetramer to the 48-mer. We found that the initial U-shaped topology per monomer is maintained over time in all oligomers. The observed deviations of protofilaments from the starting structure increase significantly with size due to the twisting of the in-register parallel 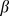-sheets. This twist causes long protofilaments to be unstable and leads to a breakage. Protofilament pairs, which are stabilized by a hydrophobic interface, exhibit more fibril-like properties such as the overall structure and the twist angle. Thus, they can act as stable conformational templates for further fibril growth. Key properties like the twist angle, shape complementarity, and energetics show a size-dependent behavior so that small oligomers favor the protofilament topology, whereas large oligomers favor the protofilament pair topology. The region for this conformational transition is at the size of approximately twelve A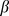 monomers. From that, we propose the following growth mechanism from A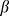 oligomers to fibrils: (1) elongation of short protofilaments; (2) breakage of large protofilaments; (3) formation of short protofilament pairs; and (4) elongation of protofilament pairs.

## Introduction

Alzheimer’s disease (AD) was first described in 1907 by the psychiatrist and neuropathologist Alois Alzheimer[1]. Histological examinations of AD brains indicate an accumulation of the Amyloid-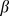 peptide (A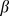) into plaques outside the neurons, leading to hyperphosphorylation of the tau protein which itself aggregates inside the neurons. A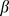 is a fragment of the ubiquitously occuring transmembrane amyloid precursor protein (APP) that is proteolytically cleaved by two secretases to yield peptides of different length, mainly 40 or 42 residues long[2], [3]. A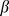 monomers exist in a dynamic equilibrium of a variety of conformations and the 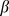-sheet form can aggregate to oligomers and higher structures. Currently, soluble oligomers of the misfolded A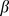 peptide are thought to be the toxic species in AD rather than amyloid fibrils in the plaques[4]. However, there is still no complete understanding of the cause of this neurodegenerative and lethal disease.

**Figure 1:**
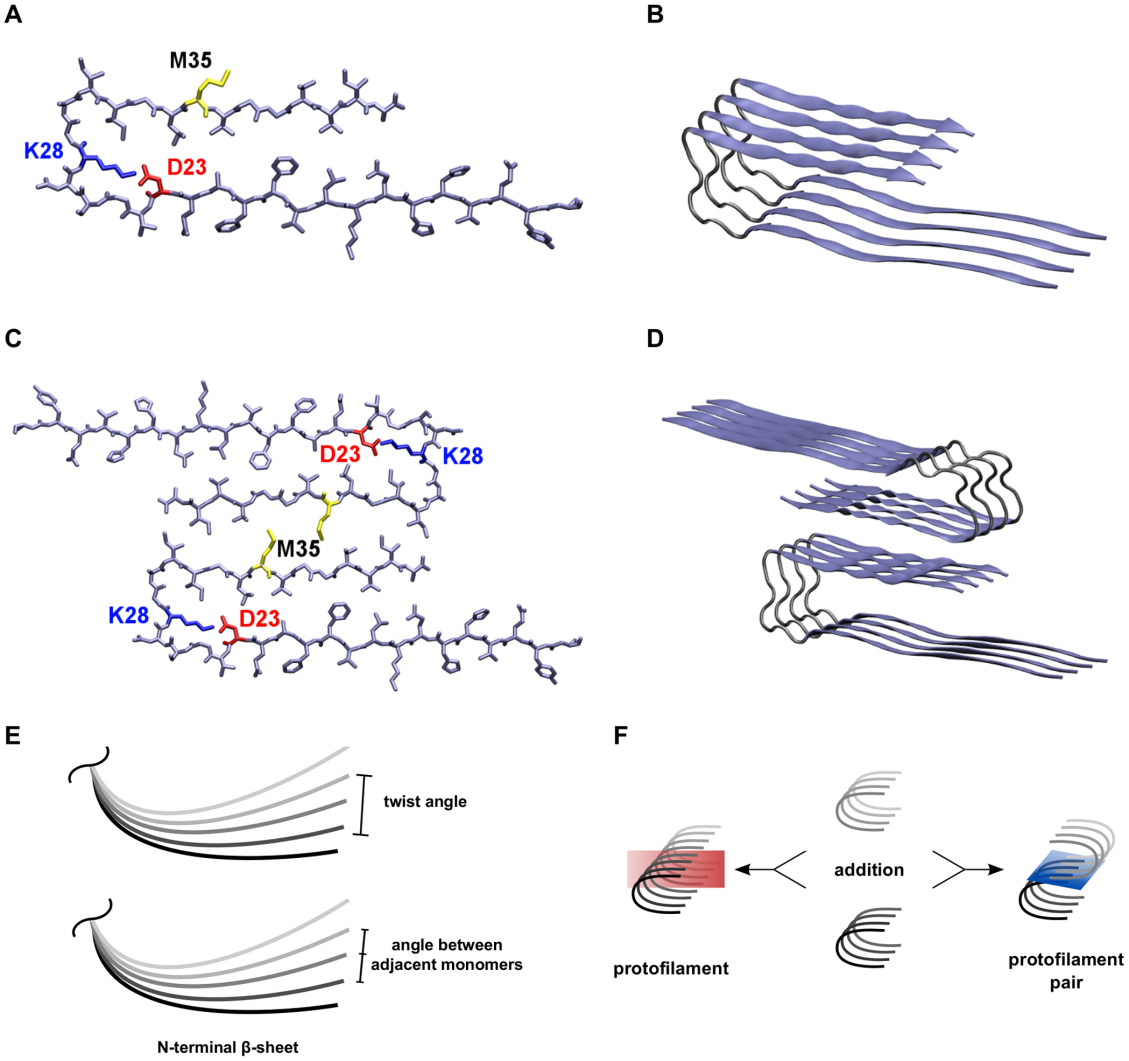
Presentation of the system and explanation of calculations. (A) The orientation of sidechains in the protofilament monomer with the salt bridge between residues D23 (red) and K28 (blue), and residue M35 (yellow) of the C-terminus pointing towards the surrounding solvent. (B) The 4-mer (O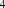) as an example for the orientation of peptide chains within the protofilaments. (C) The interaction between hydrophobic residues around M35 (yellow) in the C-termini of two opposite protofilaments constitutes the interface in the protofilament pairs. (D) The 8-mer (O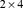) as an example for the orientation of peptide chains in the protofilament pairs. (E) Two different angles were analyzed, the twist angle and the angle between adjacent monomers. (F) Two oligomers can either be combined to form a longer protofilament (elongation) or be merged via C-terminal contacts to form a protofilament pair (thickening). Therefore, two types of MM/GBSA calculations were performed: segmentation of protofilaments along the red plane and segmentation of protofilament pairs along the blue plane.

In fibrils and fibrillar oligomers, the A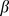 monomer adopts a U-shaped topology (Figure 1A) due to an overall sheet-turn-sheet structure identified by NMR techniques[5]–[8]. The hydrogen bond formation between two adjacent monomers in the stack results in a cross-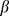 structure that is also known from various other aggregating peptides[9]–[13]. Additional stabilizing effects arise from the bifurcated salt bridge between D23 and K28 and from interactions within the central hydrophobic core consisting of amino acids F19, A21, I32, L34, and V36[5], [7], [8].

The growth of a fibrillar A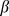 structure occurs in two major processes. First, the addition of A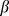 chains onto the ends of the fibrillar oligomer, i.e. a protofilament, is called elongation and occurs along the protofilament axis (Figure 1B). Second, the lateral merging of two such protofilaments into a pair is called thickening and occurs parallel to the protofilament elongation axis (Figure 1D)[14]. Although each of the two 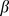 strands of the protofilament may serve as contact interface for thickening, there is experimental evidence for the presence of a CC-interface in A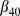 and A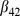 with key residues I31, M35, V39, and I41 (Figure 1C)[15]. Additional theoretical studies confirm the importance of the hydrophobic CC-interface for protofilament thickening[16], [17]. Elongation and thickening are two competing processes that are difficult to dissect experimentally due to conformational heterogenicity of oligomers, concomitant presence of different oligomeric states, and low solubility of higher oligomers.

Molecular dynamics (MD) simulations have proven to be potent tools in revealing structural properties and aggregation behavior of A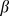 (see e.g. [18]–[20]). Energetical and structural stabilities of protofilaments with five A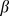-strands and protofilament pairs with ten strands have been studied in the group of Nussinov[16]. Buchete et al. investigated protofilament pairs with eight and twelve A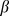 monomers[21], [22]. The two growth mechanisms have further been investigated using MD simulations; the addition of monomers to already formed fibrils is a thermodynamically driven process[23]–[25], whereas the formation of multiple layered protofilament pairs by fibrillar oligomers is kinetically more favored, i.e. the formation of protofilaments is a prerequisite for the formation of protofilament pairs[26]. As fibrils can be reservoirs for toxic oligomers[4], [27], further insight into early fibril growth is important. Previously, we have studied the U-shaped topology in fibrillar A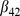 monomers to pentamers[28]. However, a systematic study about the conformation of larger oligomers is missing.

In this contribution, we extend our work on fibrillar oligomers[28] towards larger A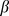 oligomers up to the 48-mer by investigating the relative stability of protofilaments and protofilament pairs using MD simulations. One particular aim was to identify the most probable size at which the transition between the two topologies occurs, i.e. when the formation of a protofilament pair becomes favored over elongation of the protofilament. Our results indicate that two A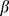 pentamers or hexamers exhibit the necessary compatibility to form protofilament pairs, so that we can propose a detailed growth mechanism for fibrillar oligomers that links the two growth mechanisms of elongation and thickening.

## Materials and Methods

As in our previous study[28], the starting structure for all systems is model 10 of PDB entry 2BEG that is an A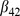 fibril structure based on NMR spectroscopic data[5]. Residues 9 to 16 were added in an extended conformation using Sybyl[29] and capped with an acetyl group. Although the N-terminal residues may play an active role in A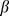 aggregation e.g. via the complexation of transition metal ions[30], [31], the N-terminus itself is not part of the fibrillar cross-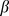 structure [5], . Residues 1–8 are therefore omitted in the simulations for the sake of consistency[28]. Hence, the sequence of a monomer is AcGY
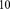
EVHHQKLVFF
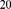
AEDVGSNKGA
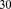
IIGLMVGGVV
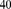
IA. The innermost chain C of the PDB model was taken as reference with a conformationally adjusted side chain of M35 to avoid clashes upon interface modeling.

All protofilament structures were created by generating idealized protofilaments of different size via a mean translation vector 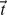 from chain B to chain D (Equation 1),
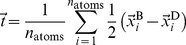
(1)where 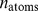 are the total number of atoms of one A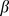 chain, and 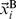 and 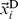 are the coordinates of the 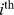 atom in chain B or D, respectively.

To obtain initial structures for the protofilament pairs, a pair of hexamers as a reference structure was set up first. Therefore, one protofilament hexamer was docked with Gramm[32] to the hydrophobic interface of the second hexamer according to the experimentally determined side chain register in A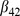 [15]. This initial model of a protofilament pair was further finetuned by short MD simulations with Amber[33]. Protofilament pairs of different length were then generated using the same strategy described above for the protofilaments (Equation 1). Figures 1B and D depict the starting structures of O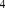 and O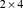 as an example for a protofilament and a protofilament pair, respectively. To distinguish between the oligomers of the two topologies protofilament and protofilament pair, we use a systematic nomenclature: tiny oligomers are O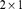, O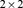, and O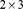, as well as O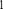 through O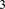 studied previously[28]; small oligomers are O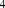, O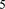, O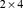, and O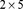; medium-sized oligomers are O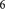 and O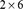; and large oligomers are O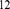, O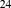, O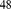, O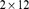, and O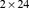.

All systems were neutralized by adding an appropriate amount of sodium counter ions and solvated within a TIP3P[34] water box with a box border distance of 15 Å (20 Å for O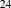 and O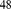). The calculation setup for all simulations can be found in Table 1. Calculations were performed using the Amber11 program suite[33] with the ff99SB force field[35]–[37] and default settings for non-bonded interactions. Long-range electrostatics were calculated with the particle mesh Ewald (PME) approximation[38], [39]; Shake was used for equilibration and simulation to constrain hydrogen atoms[40].

**Table 1:** Calculation setup of the different oligomer simulations.

system	charge	totalatoms	watermolecules	box dimensions (x, y, z [Å])		
O_4_	–8	35,018	10,990	60.3	69.7	103.3
O_5_	–10	38,671	12,037	67.2	69.2	103.3
O_6_	–12	44,400	13,776	71.5	69.2	111.3
O_8_	–16	48,994	14,966	79.5	73.0	104.4
O_10_	–20	56,336	17,072	93.7	71.0	104.4
O_12_	–24	64,140	19,332	103.3	71.8	105.3
O_24_	–48	198,930	62,214	172.4	96.9	134.9
O_48_	–96	352,935	109,453	303.8	96.9	134.9
O_2×1_	–4	92,044	30,340	octahedron*		
O_2×2_	–8	91,766	29,902	octahedron*		
O_2×3_	–12	92,436	29,788	octahedron*		
O_2×4_	–16	63,370	19,758	92.2	128.1	65.7
O_2×5_	–20	69,341	21,407	92.2	128.1	71.7
O_2×6_	–24	79,743	24,533	94.3	128.1	78.9
O_2×12_	–48	124,914	37,542	99.8	130.6	114.2
O_2×24_	–96	216,102	63,842	101.9	146.0	170.0

* The different shape of the solvent box accounts for the anticipated large conformational flexibility.

Three consecutive minimization steps with decreasing restraints were carried out with 5,000 cycles each. After 2,500 cycles the minimization method was switched from steepest descent to conjugate gradient. In the first step, the peptide atoms except hydrogens were harmonically restrained, in the second step, only the peptide heavy atoms were held fixed using a force constant of 10 kcal ⋅ mol
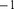
Å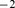 for both steps. In the third step, no restraints were applied. The system was then gradually heated to the target temperature (310 K) and the water density was kept at 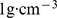. The system was held at constant pressure for 0.1 ns with small restraints on all heavy atoms; next, restraints were reduced to backbone atoms only for 0.4 ns. Both times a force constant of 5 kcal  mol
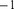
Å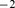 was used. After that, 0.5 ns without any restraints were simulated. The ensemble was switched to NVT and the system was now kept at constant temperature and constant volume. For the systems O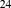 and O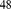 two independent simulations were conducted for verification. A timestep of 2 fs was chosen for all simulations and snapshots were collected every 5 ps. In total, 16
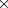
50 ns were simulated saving 10,000 snapshots for each system.

The twist angle of the whole system was calculated with the analysis program ptraj (integrated in Amber11) via the dihedral angles of the C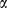 atoms of V18 and V24 of the second and the penultimate monomer. The angle of two adjacent monomers is defined as the angle between the position vectors of residues V18 and V24 of the monomers in analogy to Zheng et al.[16] (Figure 1E). Analysis of the water channel was carried out with Mole[41]. The program Dssp was used for the calculation of secondary structure elements[42], [43]. For calculating the shape complementarity the program Sc from the suite CCP4 was used[44], [45]. Salt bridges were monitored via the distance between the carboxylic oxygens of D23 and the ammonium nitrogen of K28 using a cutoff of 4.2 Å[28]. Energetic analyses were performed using the MM/GBSA method[46] from Amber11 (Generalized Born model 2[47]). To obtain the stabilization for the two mechanisms of elongation and thickening, interaction energies within symmetrically segmented protofilaments and protofilament pairs were calculated (Figure 1F). Visualization of the trajectories, analysis of the hydrogen bonds, and generation of structure images were carried out with Vmd[48]. All simulations were performed on the compute cluster of the “Regionales Rechenzentrum Erlangen”.

## Results and Discussion

### Overall Structural Stability

**Figure 2:**
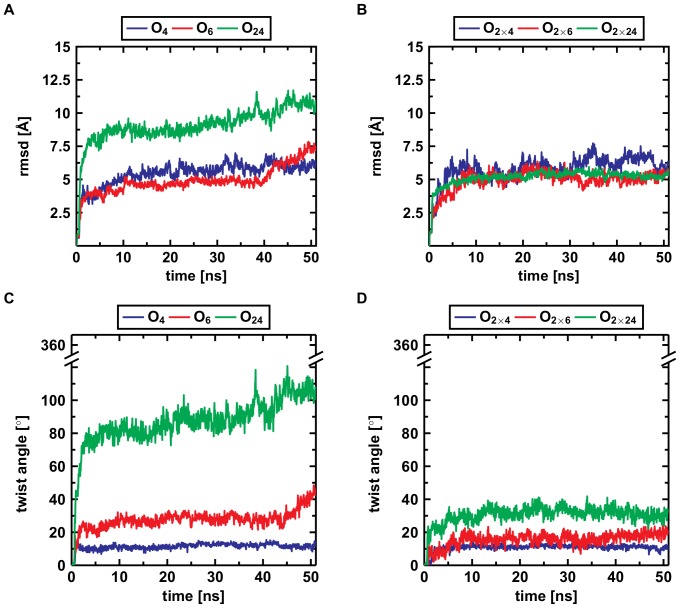
Rmsd values and twist angle for a small, medium,and large protofilament and its corresponding protofilament pair. The rmsd values for the protofilaments (A) increase significantly with size of A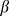 oligomers. Upon formation of the C-terminal interface leading to protofilament pairs (B), the rmsd shows no difference between the small, medium or large system. Parallel in-register 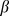-sheets reveal a general twist along the growth axis. The twist angle increases with size in the protofilaments (C) and is the reason for the rather high rmsd values (A). Upon formation of the C-terminal interface leading to protofilament pairs (D), the twist angle remains stable over time, indicating that addition of a second layer counteracts twisting of parallel 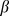-sheets.

**Figure 3:**
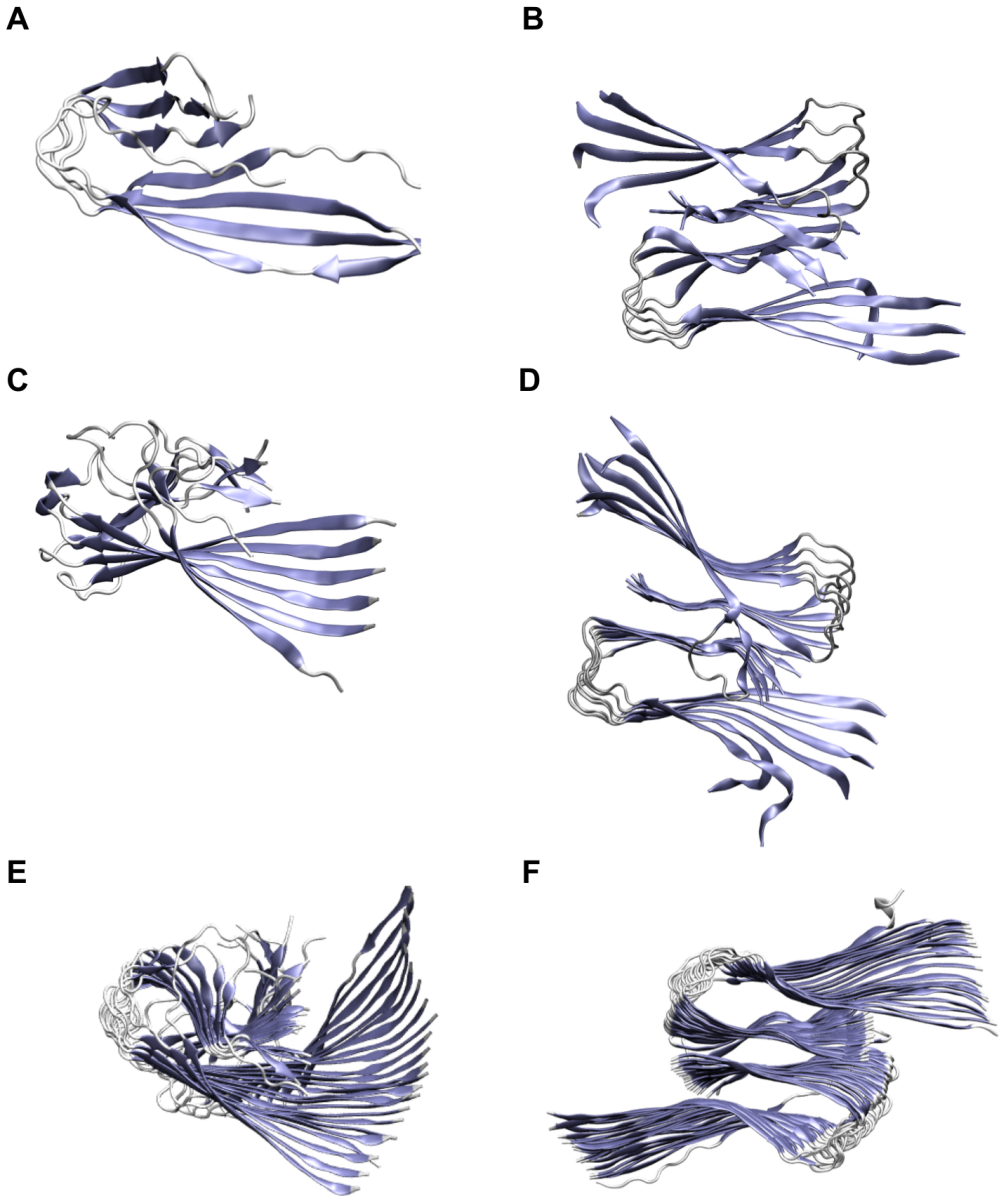
Final structures of the simulations of a small, medium, and large protofilament and its corresponding protofilament pair. (A) The protofilament tetramer (O_4_) reveals a large twist angle and a flexible hinge region in the C-terminus. (B) The protofilament pair octamer (O_2×4_) shows a similar twist angle to the O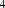, but the hydrophobic residues in the C-terminus are covered by the second layer. (C) The protofilament hexamer (O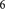) displays the large twist of the parallel 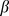-sheets. (D) The protofilament pair dodecamer (O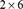) has a smaller twist angle than the protofilament hexamer due to the conteracting stabilization by the C-terminal interaction. (E) The protofilament 24-mer (O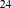) shows a small angle between adjacent monomers but the large overall twist angle. (F) The protofilament pair 48-mer (O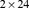) shows that the overall twist angle is reduced upon C-terminal interaction.

The root-mean-square deviation (rmsd) is an established measure in molecular dynamics studies for a quantitative analysis of conformational stability. Table 2 lists the rmsd values for all systems after 50 ns (see also Figure S7 and S8 in File SI). The smallest deviations are observed for the small protofilaments and the large protofilament pairs (Figure 2, Table 2). In contrast, the tiny protofilament pairs and the large protofilaments undergo larger structural changes (Figures 3E and S4 in File SI). However, visual inspection (Figure 3) shows that these high rmsd values do not generally result from an unfolding of the structure but rather from a systematic distortion (Figures 3E and S4–S6 in File SI).

**Table 2:** Values of the rmsd after 50 ns and the mean twist angle over the last 10 ns of the simulation.

oligomer	rmsd [Å]	twist angle [°]	angle between adjacent monomers [°]
O_4_	6.14	11.86	14.89
O_5_	8.01	22.93	13.24
O_6_	7.21	33.60	14.84
O_8_	5.26	22.35	6.08
O_10_	6.87	43.65	8.07
O_12_	7.86	62.77	9.94
O_24_	10.19	102.94	5.43
O_48_	24.34	312.19	6.17
O_2×1_	16.57	–*	–*
O_2×2_	12.35	–*	–*
O_2×3_	12.59	–*	–*
O_2×4_	6.02	10.88	12.39
O_2×5_	5.07	20.55	11.81
O_2×6_	5.61	18.82	6.20
O_2×12_	3.99	20.45	2.72
O_2×24_	5.22	30.45	2.54

* The tiny oligomers O_2×1_, O_2×2_, and O_2×3_ were excluded from systematic analysis due to loss of their initial fold.

To avoid a misinterpretation of the rmsd values, we performed a careful visual analysis of all trajectories (Figure S1–S6 in File SI). A first finding was that all three tiny protofilament pairs O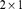, O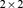, and O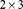 lost their initial protofilament pair conformation. The dimer O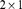 quickly collapsed into a coil structure; the C-terminal interface around M35 between the two chains opened up, and instead formed an antiparallel 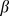-sheet around the central hydrophobic core residues (L17, F19, A21) in both chains. This newly established secondary structure element was stable throughout the simulation (Figure S4 in File SI). In the O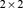 oligomer, the two fibrillar dimers retained their general fold, but again the hydrophobic CC-interface contact between the protofilament pairs was lost. However, rotating of the two dimers towards each other formed an antiparallel 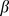-sheet between the C-terminal 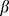-sheets. In contrast, the N-terminal sheets of both dimers did not come close enough during the simulation to form a second antiparallel 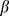-sheet (Figure S4 in File SI). The protofilament pair of the trimer O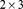 displayed a strong shear movement between the two A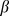 stacks. Although the two trimers stayed in contact via the hydrophobic interface throughout the simulation, they showed a certain drift along this interface. In summary, the two halves of O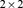 and O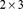 retained their general fold, whereas the two monomers in O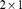 refolded completely; this behavior is very similar to the isolated systems O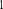, O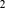, and O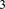 investigated previously[28]. We can therefore conclude that the hydrophobic CC-interface contact in three tiny oligomers O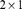, O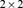, and O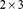 is not sufficient to stabilize the protofilament pair fold; thus, no further quantitative analyses were performed for these species.

All other A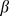 oligomers of both topologies retained the general characteristics of their initial conformation (Figure S1–S6 in File SI). Although the outer chains and the turn regions displayed an enhanced flexibility, the sheet-turn-sheet topology was stable in all chains of all oligomers and the hydrophobic interface stayed intact throughout the simulations. In the C-terminal region of the protofilaments, however, the chains bent upwards starting at residues G37/G38 to hide the hydrophobic side chains of residues 35, 39, 40, and 41 orientated towards the solvent. This flexible hinge, which was already described for small fibrilar A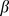 oligomers[28], is also present to a certain extent in the small protofilament pairs O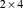 and O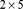. There, the flexible region disrupts the C-terminal 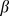-sheet, although the respective residues are actually covered by a second layer of A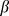 stacks.

**Figure 4:**
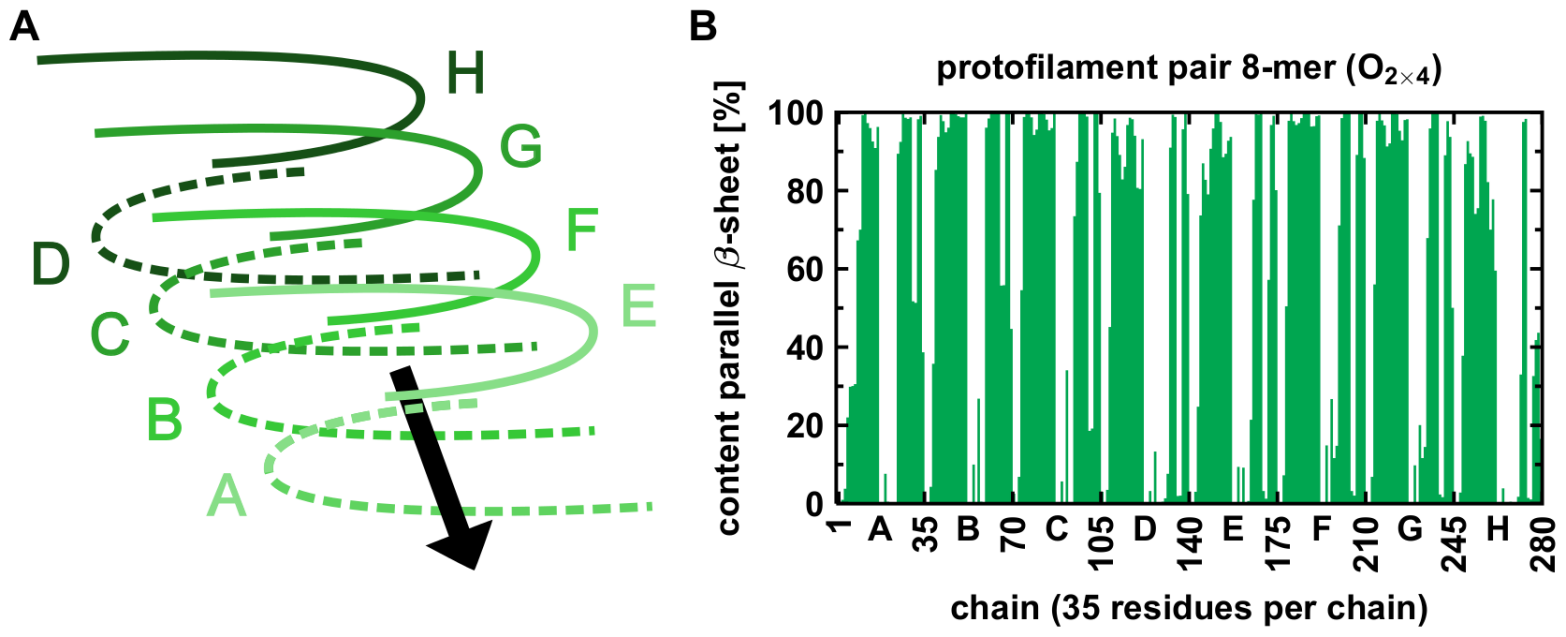
Stability of 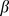-sheets in the protofilament pair 8-mer (O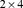). (A) Chains A and E are the terminal chains in growth-direction, chains D and H are the terminal chains on the other end of the oligomer; each chain consists of 35 residues. (B) Mean content of parallel 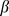-sheets. N-terminal and C-terminal 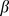-sheet of each monomer are separated by a turn region. See Figures S13–S22 in File SI for the results of all other oligomers.

The findings from visual analysis are confirmed by an analysis of the secondary structure content. Figure 4 presents the mean 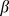-sheet content per residue for O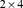 as example. Each monomeric chain displays a similar fold in the system. Small differences between the terminal chains, however, suggest different tendencies for elongation[23]–[25]. In general, these results are also found in all other oligomers; the respective plots are given in (Figures S13–S22 in File SI).

To further quantify our findings, we performed a hydrogen bond analysis. The results show that the number of hydrogen bonds converges to a constant value for each system (Figures S9 and S10 in File SI). In the case of O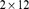 and O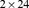, the hydrogen bonds of the parallel C-terminal strands become more stable. The decrease of hydrogen bonds in the protofilaments on the other hand is partially caused by the flexibility of the outer chains.

### Hydrated Salt Bridges

According to NMR data, the salt bridge between residues D23 and K28 is bifurcated in A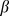 fibrils and forms intramolecular and intermolecular contacts simultaneously[5], [6], [49]. The intramolecular salt bridge contact stabilizes the U-shaped A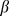 chain within the oligomer. In our simulations, these stabilizing contacts occured more frequently in the center of the oligomers than in the flexible end regions. The intermolecular salt bridges that add to the stability of the overall oligomer fold show similar characteristics. However, the occurencies at the growing end exceed those at the opposite end of the fibrillar structures, both within a single layer protofilament and in each layer of a protofilament pair. Furthermore, the additional fibrillar layer increases the stability of intermolecular salt bridges. In O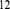 and O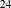, the salt bridge stability between the inner chain pair H–I and J
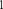
–K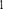, respectively, is significantly decreased, whereas no such behavior was observed in O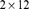 and O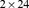. Comparing the absolute occurrencies of intra- and intermolecular salt bridges, the latter are more stable, especially for larger oligomers. Table S1 in File SI provides a detailed list of all intra- and intermolecular salt bridge contacts and their occurrencies. Our analysis confirms the structural importance of the D23–K28 salt bridge in fibrillar A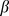 structures of different size.

**Figure 5:**
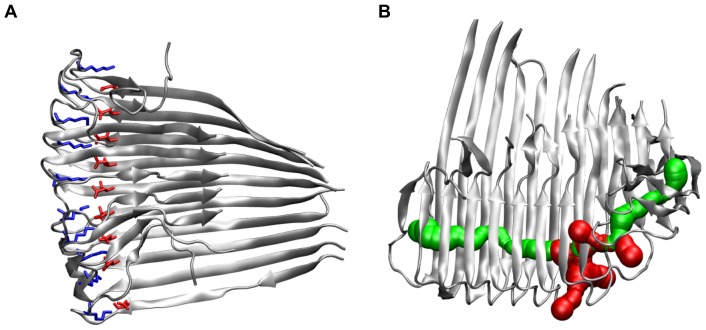
Hydration of oligomers along the salt bridges between D23 and K28. (A) Salt bridges along the 10-mer (O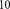), the side chains of D23 and K28 are depicted as red and blue sticks, respectively. (B) Visualization of the water channel in the protofilament 12-mer (O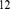, 50 ns); main entrance channel (green) and channel exits through neighboring turns (red).

Each salt bridge points towards the interior of the turn and forms a ladder of ionic interactions between adjacent A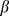 monomers (Figure 5A). Together, they span a channel along the oligomer accessible for the surrounding solvent; visual inspection confirmed water influx in all oligomers. Closer investigations showed water channel exits not only at both fibrillar ends but also between neighboring turns in oligomers of all sizes (Figure 5B). Due to their intrinsic flexibility, smaller oligomers have more channel exits than larger oligomers with tighter packed 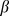-sheets. Thus, the water channel is stabilized by increasing oligomer size.

Calculations of the channel diameter (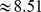 Å) and the diffusion coefficient for channel water (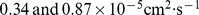 of O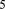 and O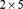, respectively) match the results of previous studies [16]. The diffusion coefficient in the channel obtained from our simulations and the one of bulk water described in literature (TIP3P water 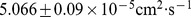 [50]) differ by one order of magnitude indicating a restricted mobility of the channel water. This effect is caused by the breakage and reformation of hydrogen bonding interactions between the water molecules and the charged amino acid side chains of D23 and K28 inside the channel.

Taken together, we observe a series of salt bridge-stabilized oligomers that all posess a water channel in accordance with the experiment and other simulations[5], [6], [16], [49]. In the next step, we wanted to investigate whether these oligomers can also provide insight into the mechanism of elongation.

### Elongation of Fibrillar Oligomers

The conformational stability of the N-terminal 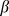-strand plays an important role in fibril elongation[23], [24]. Here, we observe that N-terminal strands are more stable in small protofilaments compared to the small protofilament pairs (Table 3). For this analysis, the N-terminal sheet is defined from G9 to F19, and the C-terminal sheet is defined from I32 to A42 (Figure 1A). The 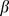-sheet content in the two different sheets increases with oligomer size in the two topologies; generally, the content of 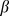-sheet is higher in the N-terminus than in the C-terminus of all protofilaments. A significant difference between the content of 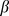-sheet in both termini in the protofilament pairs is not observed (Figure 4). The presence of 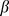-sheet in the oligomers is similar to fibrils[51], [52] and the findings of a stable 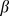-sheet at residues 9 to 19 in this work are in accordance with experimental results[6], [53], [54]. These experiments also propose that the N-terminal strand represents the initial site of recognition for an incoming A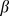 strand. The dock/lock-mechanism[23]–[25] investigated by computational means supports our observations that deposition of new A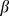 monomers can already occur on small protofilaments: the 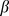-sheet content is higher in the N-terminus than in the C-terminus and parallel 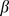-sheets are more stable in the innermost monomers of the protofilament.

**Table 3:** Mean content of parallel 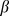-sheet (in %) and the results of the MM/GBSA calculation (in kcal

mol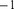).

Oligomer	whole protein	in the N-terminus	in the C-terminus	interaction energy
O_4_	50.04	77.12	45.80	–*
O_5_	48.10	76.13	36.97	–*
O_6_	53.54	83.82	49.18	–*
O_8_	60.24	85.68	56.97	–131.26
O_10_	56.01	83.63	50.67	–122.10
O_12_	60.24	82.35	63.14	–141.61
O_24_	66.37	87.81	73.52	–96.31
O_48_	67.27	87.03	78.73	–35.49
O_2×4_	60.90	53.59	45.64	–80.72
O_2×5_	60.78	59.60	53.95	–117.12
O_2×6_	63.00	62.50	61.28	–150.84
O_2×12_	68.74	77.14	79.02	–266.96
O_2×24_	71.19	86.67	86.79	–565.28

* The small oligomers O_4_, O_5_, and O_6_ were excluded from energetical analysis because no protofilament pair complement existed in this study.

Additionally, the salt bridge between D23 and K28 is important during fibril formation[6], [18], [49]. In our simulations, intramolecular salt bridges are most stable in the interior of the oligomers, whereas intermolecular salt bridges are more stable at the growing end of the oligomers, i.e. where the 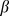-sheet content is increased (Figure 5). These findings suggest that intramolecular salt bridges are needed to stabilize the oligomer itself, while elongation of oligomers depends on stable intermolecular salt bridges at the growing end.

Thus, our results support the elongation mechanism proposed by Tarus et al.[55]: after N-terminal recognition of a new A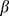 monomer at the edge of the fibril, formation of the intermolecular salt bridge between D23 and K28 can establish the turn structure which itself facilitates the formation of the U-shaped topology.

### Preference for Protofilaments or Protofilament Pairs

From the data above, both protofilaments and protofilament pairs appear elongation competent. N-terminal strands are more stable in protofilaments; therefore, the initial growth most probably occurs in this species. Since mature fibrils consist of protofilament pairs, the question arises at which size the protofilaments recombine to form protofilament pairs. In the present set of A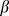 oligomers, three different properties were therefore analysed: the stabilization energy; the twist angle; and the shape complementarity.

**Figure 6:**
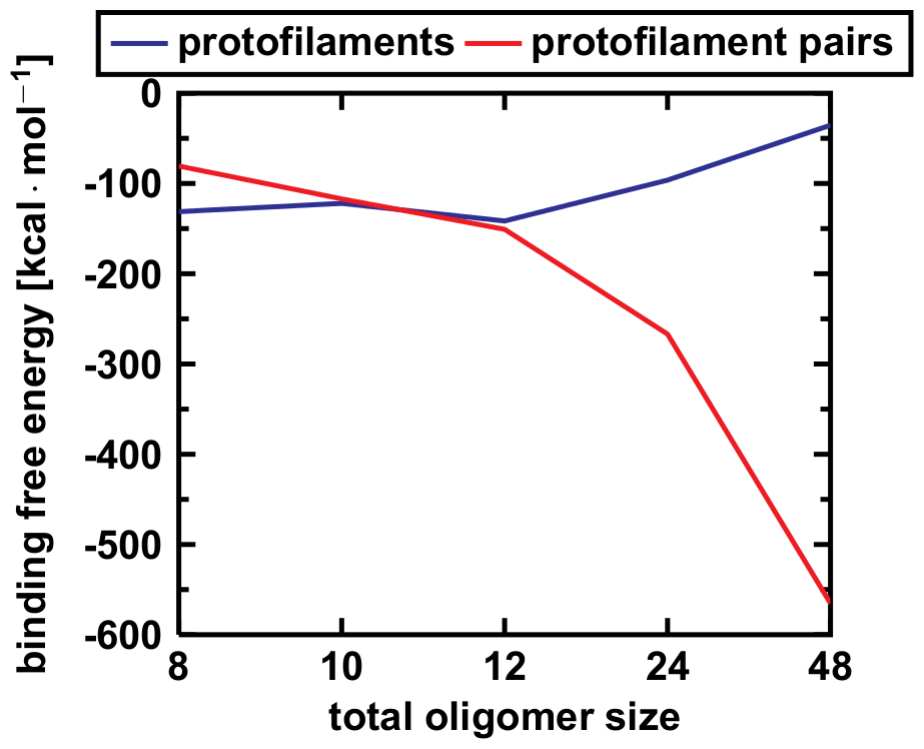
Interaction energy analysis of the protofilaments and protofilament pairs from the 8-mer to the 48-mer. The MM/GBSA interaction energy of the protofilament pairs decreases significantly with size, whereas longer protofilaments become increasingly unstable. Formation of protofilament pairs over formation of longer protofilaments becomes favored for oligomers consisting of 12 monomers. Further elongation of protofilaments increases instability.

First, we performed MM/GBSA calculations on protofilaments and protofilament pairs with 8, 10, 12, 24, and 48 monomers to find out which conformation is energetically preferred upon combining two smaller oligomers of half the size. For this analysis, protofilaments were segmented in the middle of the longitudinal growth axis and protofilament pairs were segmented along the C-terminal interface to monitor the interaction energy gained through recombination via the respective interface (Figure 1F). The energetic calculations (Table 3) reveal that up to the decamer small protofilament pairs are less favored than protofilaments. For example, the interaction energies of O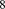 and O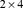 are 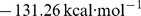 and 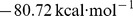, respectively; elongation is therefore preferred over thickening at this oligomer size. In contrast, the interaction energies of O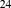 and O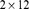 are 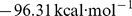 and 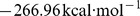, respectively, clearly favoring the growth via thickening. Additionally, the C-terminal sheets are stabilized by stacking interaction of hydrophobic surfaces which is reflected in the amount of secondary structure elements that is higher in the protofilament pairs. Taken together, we observe a distinct crossing point of the two curves where the conformation of protofilament pairs becomes energetically more stable than the conformation of the protofilament (Figure 6). Oligomers larger than 2×6 monomers, thus, should prefer the protofilament pair conformation (Table 3).

**Figure 7:**
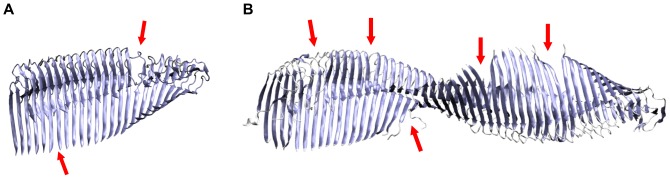
Breakage of the protofilament 24-mer (O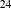) and 48-mer (O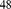). Final snapshots of the MD simulation after 50 ns show that the overtwisting of parallel 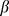-sheets initiates a breakage into oligomers of smaller size. Breakage points in the 24-mer (A) and the 48-mer (B) are indicated with red arrows.

Second, we measured the development of the twist angle because in-register 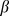-sheets generally twist around their longitudinal axis[56]. Large A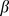 protofilaments reveal a large overall twist in MD simulations that results in a high deviation from the starting structure. We observed this behavior already in a previous study of an A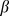 nonamer that showed an angle between adjacent monomers of approximately 5 giving rise to a total twist angle of ca. 40 [57]. In the present work, the large protofilament O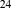 exhibits a twist greater than 100 (Table 2 and Figure 7A); the twist of O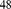 of more than 310 is depicted in Figure 7B. In contrast to the twist angles of the protofilaments that increase rapidly with length, the twist angles of the protofilament pairs increase rather slowly with increasing size (Table 2, Figure S11 and S12 in File SI). The opposite trend is observed for the angles between two adjacent monomers; with increasing oligomer size the angle decreases in both the protofilaments and the protofilament pairs (Table 2). A closer look at the small oligomers shows that the twist angles are rather similar for O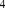 and O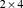, O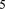 and O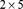, and O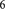 and O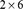; additionally, the angles between two adjacent monomers are similar for O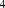 (14.89) and O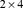 (12.39), and O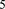 (13.24) and O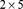 (11.81). Comparison of the angles between the other protofilaments and their protofilament pairs shows less compatibility. Therefore, the twist angle analysis is in line with the energetic results about the number of monomers that can either form a single protofilament or a protofilament pair: 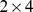 and 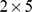 monomers fit nicely and 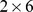 monomers should still be tolerated.

Finally, we calculated the shape complementarity for all protofilament pairs to have a measure for the steric zipper quality of the C-terminal interface, i.e. the mutual orientation of the amino acid side chains. The shape complementarity for O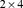 (0.673) and O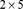 (0.715) is in the range of antibody/antigen interfaces (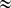 0.68)[44], whereas it is in the range of protein/protein inhibitor interfaces (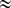 0.74)[44] for the larger protofilament pairs (O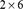: 0.742, O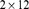: 0.733, O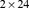: 0.744) which is in line with previous studies[9], [10], [16]. The low degree of complementarity in the small oligomers might be due to the presence of water molecules in O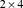 and O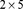 near the flexible G37/38 hinge in the interface; water along the fibril interface was already detected in other simulation studies[22], [58]. Conversely, O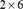 already exhibits a high shape complementarity that does not further increase for the larger O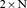 oligomers.

The stabilization energy, the angle between two adjacent monomers, and the shape complementarity are indicators that stable protofilament pairs can be formed from small protofilaments. Protofilament tetramers and pentamers can readily associate to the protofilament pair structure without any changes in the twist angle, as can be seen from similar angles between two adjacent monomers and a similar shape complementarity. For the protofilament hexamer O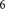 and protofilament pair dodecamer O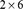 the angle between two adjacent monomers starts to diverge; the interface of the protofilament pair strengthens due to more hydrophobic interactions resulting in a higher shape complementarity and a reduced twist of 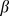-sheets compared to the protofilament hexamer. According to the data above, the favorable size of a protofilament that can combine to form a protofilament pair seems to be 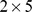 to 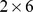 monomers.

### Instabilities in Large Protofilaments

Next, we investigated to what extend large protofilaments retain their general topology. The values of the twist angles for the large protofilaments O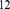 (62.77), O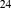 (102.94), and O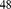 (312.19) show that strong deviations from the starting structure result from the twisting of the in-register parallel 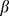-sheets as described in literature[12], [56]. The overtwisting of the protofilaments (6.17 per monomer in the 48-mer compared to 0.45 per monomer in the fibril[5]) results in a breakage of the 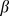-sheets (Figure 7). Additionally, clearly reduced intermolecular D23–K28 salt bridge stabilities for inner chains in these species are also indicative of such structural instabilities (Table S1 in File SI). This breakage was also observed in experiments with oligomers[59] and other computational studies of protofilament dodecamers[60]; in all cases, the fragments are of the sizes from 4 to 9 monomeric subunits. Moreover, two energetical effects can be seen in Figure 6. First, large protofilaments are less stable compared to their protofilament pair counterparts, and second, protofilaments become more instable for increasing size. Compare the values for O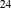 (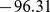) and O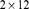 (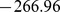); O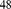 (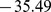) and O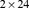 (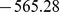), and, to a lesser extent, O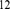 (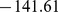) and O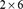 (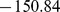)(all values in 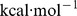).

Protofilaments exhibit a stable N-terminal strand and can therefore grow via elongation[23]–[25]. Thickening, i.e. the lateral association of a second layer, is kinetically less favorable and occurs on larger timescales[26], [59]. Further elongation of protofilaments leads to a breakage producing more growing ends that might explain the exponential growth and the spread throughout the brain[59]. When protofilament fragments break off a fibril, they can exert their neurotoxic properties or recombine to form protofilament pairs. The latter ones are conformationally stable and are expected to allow fast fibril growth, i.e. not staying at the O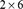 size.

Our simulations suggest that oligomers larger than the dodecamer do not exist *in vivo*. They either break apart if they are further elongated in their protofilament conformation or combine to protofilament pairs that readily grow to fibrils. These observations are in agreement with experiments that mainly observe oligomers the size of dodecamers[61]–[64]. Other computational studies proposed a similar smallest number of A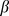 monomers to be stable in protofilament pair conformation as suggested here[65], [66].

### Protofilament Pairs are Fibril Primers

Several studies indicate that fibril growth must occur via thickening[5], [7], [15], [16], [19]. The protofilament pairs in this study exhibit certain fibrillar properties suggesting that they are precursors or primers of large A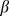 fibrils. The structures of A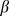 fibrils reveal that the fibril is twisted around its axis[5], [12] which is also observed for the oligomers. In fibrils, the twist angle is 0.45 per monomer[5], whereas it is 2.45 per monomer for O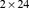 here. As the twist angle per monomer decreases with length in the protofilament pairs, they might match the fibril value of 0.45 for very long protofilament pairs. The twist of protofilament pairs is much more similar to the fibril when compared to protofilaments (O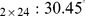, O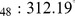). The twist angle in protofilament pairs is smaller than in protofilaments (Table 2) because the hydrophobic interaction via the C-terminal 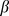-sheet stabilizes the protofilament pairs. Additionally, protofilament pairs show a good shape complementarity and exhibit a steric zipper with the side chains of the C-terminal interface interacting via van der Waals contacts[9], [10], [16], [19], [67].

Concerning toxicity, oligomers are more harmful than fibrils[68]–[70]. As protofilament pairs exhibit fibril-like properties, it can be assumed that they are less toxic than small protofilaments. Large protofilament pairs can act as nucleation points for fibril growth, because they possess a stable and fibril-like topology. In constrast, we observed an instability of large protofilaments apparent in several breaking-points. They can therefore act as reservoirs for small toxic oligomers and can seed further growth of protofilaments.

### Proposed Growth Mechanism of Fibrillar Oligomers

**Figure 8:**
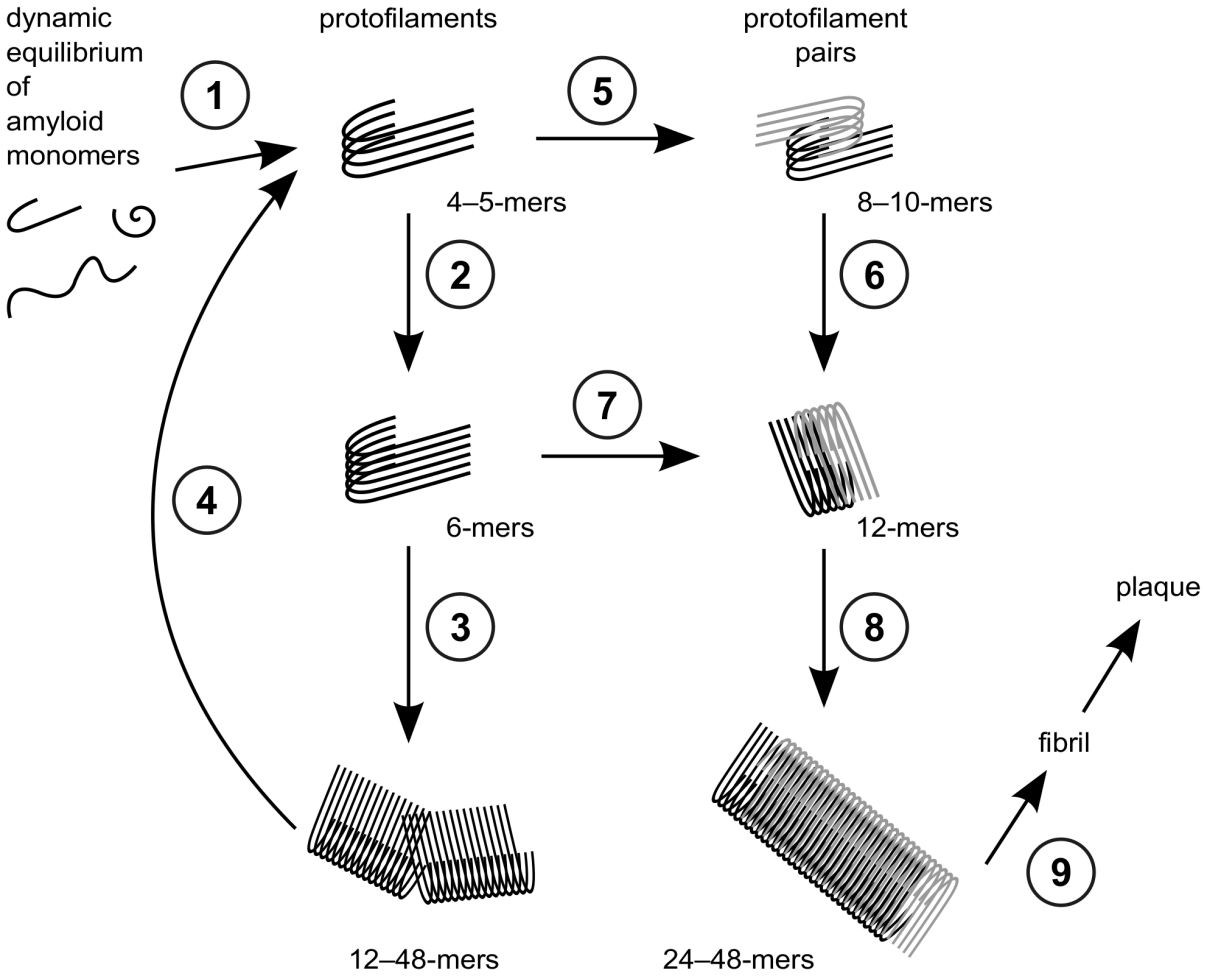
Model for the growth mechanism of fibrillar oligomers. Elongation of protofilaments and thickening to protofilament pairs can occur in the following steps: (1) aggregation of monomers forming small protofilaments; (2) and (3) elongation of protofilaments along the longitudinal growth axis; (4) fragmentation of large protofilaments into small protofilaments; (5) merging of two small protofilaments leading to a small protofilament pair; (6) addition of monomers elongating small protofilament pairs; (7) merging of two medium protofilaments leading to a medium protofilament pair; (8) addition of monomers elongating medium protofilament pairs; and (9) downstream aggregation of A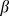 via elongation of large protofilament pairs to fibrils and plaques.

A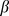 aggregation is a complex process that can be influenced by the surrounding microenvironment like metal ions concentration[71], [72] or pH changes[73], [74]. To shed more light onto the mechanism of oligomer growth, all-atom molecular dynamics simulations were used to study the conformational stability of A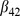 protofilaments and protofilament pairs in a systematic way. From our results, a general growth mechanism combining the existing mechanisms of elongation and thickening can be proposed. Herein, the preferred oligomer growth mechanism changes from elongation to thickening at the size of decamers and dodecamers, as depicted in Figure 8.

The formation of a fibrillar oligomer seed, i.e. a small protofilament, from the pool of monomers is the prerequisite for fibril nucleation (No. 1 in Figure 8). For the elongation via addition of monomers, stable N-terminal 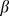-sheets are required according to the dock/lock-mechanism[23]–[25]. Analysis of secondary structure elements revealed stable 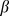-sheets in the N-termini and higher content in the protofilaments than in the small protofilament pairs (Figure 3). Therefore, protofilaments of all size, the large protofilament pairs, and, to a lesser extent, the small protofilament pairs can be elongated along the longitudinal growth axis (No. 2, 3, 6, and 8 in Figure 8).

As parallel 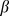-sheets twist around their axis, the twist angle was measured for all oligomers (Table 2). In protofilament pairs, the angle increases slowly with size, whereas the large protofilaments reveal a large twist that results in breakage sites within these instable oligomers (Figure 7). This instability was further confirmed by energetic analyses (Figure 3) that showed a decrease of stability for the protofilaments. In contrast, the protofilament pairs gain stability upon longitudinal growth. Therefore, large oligomers become structurally instable and break into smaller protofilaments (No. 4 in Figure 8).

Distortion and structural instabilities of large protofilaments hamper their recombination to protofilament pairs, so small protofilaments need to form the protofilament pairs. The comparison of the angles between two adjacent monomers (Table 2) in the small protofilaments O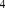 and O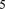 and their corresponding protofilament pairs O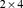 and O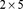 reveals matching values. Thus, two of those small protofilaments can readily dimerize to protofilament pairs (No. 5 in Figure 8) and need no structural reorganization before dimerization, although the energetic gain is rather small. For larger A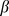 oligomers, the dimerization of two protofilaments to protofilament pairs is driven by the gain of interaction energy. While the twist angle disfavors the dimerization of larger protofilaments, it is still rather similar between O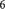 and O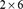. Furthermore, larger protofilament pairs from the O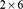 onward show a high shape complementarity. Therefore, two protofilament hexamers have a favorable size to form a protofilament pair dodecamer (No. 7 in Figure 8).

The energetic MM/GBSA analysis (Figure 6) revealed the dodecamer as crossing point for the stability of the two topologies. Up to 12 A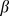 monomers, the protofilament conformation is preferred, whereas the protofilament pair conformation is energetically more favorable for large oligomers. Therefore, the growth mechanism changes from elongation of protofilaments to thickening, i.e. formation of protofilament pairs. Further downstream, protofilament pairs can grow via elongation and further thickening to form fibrils and finally plaques (No. 9 in Figure 8).

In summary, we propose the following growth mechanism from A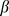 oligomers to fibrils: first, short protofilaments grow via elongation; second, large protofilaments become structurally instable and break apart; third, short protofilaments combine to short protofilament pairs; and finally, protofilament pairs grow further by elongation. Thus, A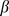 protofilament pairs can act as seeds for fibril formation, whereas protofilaments behave as templates for the addition of further monomers and therefore might be responsible for toxicity.

## Supporting Information

File S1 **Figures S1–S22 & Table S1.**
(PDF)

## References

[1] Alzheimer A (1907) Über eine eigenartige Erkrankung der Hirnrinde. Allg Zschr Psychiat 64: 146–148.

[2] Hardy J, Selkoe DJ (2002) The amyloid hypothesis of Alzheimer’s disease: progress and problems on the road to therapeutics. Science 297: 353–356. 12130773 10.1126/science.1072994

[3] Selkoe DJ (2004) Cell biology of protein misfolding: the examples of Alzheimer’s and Parkinson’s diseases. Nat Cell Biol 6: 1054–1061. 15516999 10.1038/ncb1104-1054

[4] Haass C, Selkoe DJ (2007) Soluble protein oligomers in neurodegeneration: lessons from the Alzheimer’s amyloid beta-peptide. Nat Rev Mol Cell Biol 8: 101–112. 17245412 10.1038/nrm2101

[5] Lührs T, Ritter C, Adrian M, Riek-Loher D, Bohrmann B, et al (2005) 3D structure of Alzheimer’s amyloid-beta(1–42) fibrils. Proc Natl Acad Sci U S A 102: 17342–17347. 16293696 10.1073/pnas.0506723102 PMC1297669

[6] Petkova AT, Ishii Y, Balbach JJ, Antzutkin ON, Leapman RD, et al (2002) A structural model for Alzheimer’s beta-amyloid fibrils based on experimental constraints from solid state NMR. Proc Natl Acad Sci U S A 99: 16742–16747. 12481027 10.1073/pnas.262663499 PMC139214

[7] Petkova AT, Yau WM, Tycko R (2006) Experimental constraints on quaternary structure in Alzheimer’s beta-amyloid fibrils. Biochemistry 45: 498–512. 16401079 10.1021/bi051952q PMC1435828

[8] Paravastu AK, Leapman RD, Yau WM, Tycko R (2008) Molecular structural basis for polymorphism in Alzheimer’s beta-amyloid fibrils. Proc Natl Acad Sci U S A 105: 18349–18354. 19015532 10.1073/pnas.0806270105 PMC2587602

[9] Nelson R, Sawaya MR, Balbirnie M, Madsen AØ, Riekel C, et al (2005) Structure of the cross-beta spine of amyloid-like fibrils. Nature 435: 773–778. 15944695 10.1038/nature03680 PMC1479801

[10] Sawaya MR, Sambashivan S, Nelson R, Ivanova MI, Sievers SA, et al (2007) Atomic structures of amyloid cross-*β* spines reveal varied steric zippers. Nature 447: 453–457. 17468747 10.1038/nature05695

[11] Sunde M, Serpell LC, Bartlam M, Fraser PE, Pepys MB, et al (1997) Common core structure of amyloid fibrils by synchrotron X-ray diffraction. J Mol Biol 273: 729–739. 9356260 10.1006/jmbi.1997.1348

[12] Esposito L, Pedone C, Vitagliano L (2006) Molecular dynamics analyses of cross-beta-spine steric zipper models: beta-sheet twisting and aggregation. Proc Natl Acad Sci U S A 103: 11533–11538. 16864786 10.1073/pnas.0602345103 PMC1544204

[13] Fändrich M, Meinhardt J, Grigorieff N (2009) Structural polymorphism of Alzheimer Abeta and other amyloid fibrils. Prion 3: 89–93. 19597329 10.4161/pri.3.2.8859 PMC2712605

[14] Stroud JC, Liu C, Teng PK, Eisenberg D (2012) Toxic fibrillar oligomers of amyloid-*β* have cross-*β* structure. Proc Natl Acad Sci U S A 109: 7717–7722. 22547798 10.1073/pnas.1203193109 PMC3356606

[15] Sato T, Kienlen-Campard P, Ahmed M, Liu W, Li H, et al (2006) Inhibitors of amyloid toxicity based on beta-sheet packing of Abeta40 and Abeta42. Biochemistry 45: 5503–5516. 16634632 10.1021/bi052485f PMC2593882

[16] Zheng J, Jang H, Ma B, Tsai CJ, Nussinov R (2007) Modeling the Alzheimer Abeta17–42 fibril architecture: tight intermolecular sheet-sheet association and intramolecular hydrated cavities. Biophys J 93: 3046–3057. 17675353 10.1529/biophysj.107.110700 PMC2025669

[17] Berhanu WM, Hansmann UHE (2012) Structure and dynamics of amyloid-*β* segmental polymorphisms. PLoS One 7: e41479. 22911797 10.1371/journal.pone.0041479 PMC3404032

[18] Ma B, Nussinov R (2002) Stabilities and conformations of Alzheimer’s beta-amyloid peptide oligomers (Abeta 16–22, Abeta 16–35, and Abeta 10–35): Sequence effects. Proc Natl Acad Sci U S A 99: 14126–14131. 12391326 10.1073/pnas.212206899 PMC137848

[19] Ma B, Nussinov R (2006) Simulations as analytical tools to understand protein aggregation and predict amyloid conformation. Curr Opin Chem Biol 10: 445–452. 16935548 10.1016/j.cbpa.2006.08.018

[20] Ma B, Nussinov R (2002) Molecular dynamics simulations of alanine rich beta-sheet oligomers: Insight into amyloid formation. Protein Sci 11: 2335–2350. 12237456 10.1110/ps.4270102 PMC2373704

[21] Buchete NV, Hummer G (2007) Structure and dynamics of parallel beta-sheets, hydrophobic core, and loops in Alzheimer’s A beta fibrils. Biophys J 92: 3032–3039. 17293399 10.1529/biophysj.106.100404 PMC1852365

[22] Buchete NV, Tycko R, Hummer G (2005) Molecular Dynamics Simulations of Alzheimer’s *β*-Amyloid Protofilaments. J Mol Biol 353: 804–821. 16213524 10.1016/j.jmb.2005.08.066

[23] Takeda T, Klimov DK (2009) Probing Energetics of Abeta Fibril Elongation by Molecular Dynamics Simulations. Biophys J 96: 4428–4437. 19486667 10.1016/j.bpj.2009.03.015 PMC2711452

[24] Takeda T, Klimov D (2009) Replica exchange simulations of the thermodynamics of Abeta fibril growth. Biophys J 96: 442–452. 19167295 10.1016/j.bpj.2008.10.008 PMC2716483

[25] Takeda T, Klimov DK (2009) Side Chain Interactions Can Impede Amyloid Fibril Growth: Replica Exchange Simulations of A*β* Peptide Mutant. J Phys Chem B 113: 11848–11857. 19708712 10.1021/jp904070w PMC2765228

[26] Wu C, Bowers MT, Shea JE (2010) Molecular structures of quiescently grown and brain-derived polymorphic fibrils of the Alzheimer amyloid abeta9–40 peptide: a comparison to agitated fibrils. PLoS Comput Biol 6: e1000693. 20221247 10.1371/journal.pcbi.1000693 PMC2832665

[27] Shankar GM, Li S, Mehta TH, Garcia-Munoz A, Shepardson NE, et al (2008) Amyloid-*β* protein dimers isolated directly from Alzheimer’s brains impair synaptic plasticity and memory. Nat Med 14: 837–842. 18568035 10.1038/nm1782 PMC2772133

[28] Horn AHC, Sticht H (2010) Amyloid-beta42 oligomer structures from fibrils: a systematic molecular dynamics study. J Phys Chem B 114: 2219–2226. 20104925 10.1021/jp100023q

[29] Tripos (1991–2008) Sybyl 7.3. St. Louis, Missouri, USA.

[30] Miller Y, Ma B, Nussinov R (2010) Zinc ions promote Alzheimer Abeta aggregation via population shift of polymorphic states. Proc Natl Acad Sci U S A 107: 9490–9495. 20448202 10.1073/pnas.0913114107 PMC2906839

[31] Parthasarathy S, Long F, Miller Y, Xiao Y, McElheny D, et al (2011) Molecular-level examination of Cu2+ binding structure for amyloid fibrils of 40-residue Alzheimer’s *β* by solid-state NMR spectroscopy. J Am Chem Soc 133: 3390–3400. 21341665 10.1021/ja1072178 PMC3074258

[32] Vakser IA (1992–2003). GRAMM: Global Range Molecular Matching.

[33] Case DA, Darden TA, Cheatham TE, Simmerling CL, Wang J, et al.. (2010) Amber 11. University of California, San Francisco.

[34] Jorgensen WL, Chandrasekhar J, Madura JD, Impey RW, Klein ML (1983) Comparison of simple potential functions for simulating liquid water. J Chem Phys 79: 926–935.

[35] Wang J, Cieplak P, Kollman PA (2000) How well does a restrained electrostatic potential (RESP) model perform in calculating conformational energies of organic and biological molecules? J Comput Chem 21: 1049–1074.

[36] Cornell WD, Cieplak P, Bayly CI, Gould IR, Merz KM, et al (1995) A Second Generation Force Field for the Simulation of Proteins, Nucleic Acids, and Organic Molecules. J Am Chem Soc 117: 5179–5197.

[37] Hornak V, Abel R, Okur A, Strockbine B, Roitberg A, et al (2006) Comparison of multiple Amber force fields and development of improved protein backbone parameters. Proteins 65: 712–725. 16981200 10.1002/prot.21123 PMC4805110

[38] Cheatham TE, Miller JL, Fox T, Darden TA, Kollman PA (1995) Molecular Dynamics Simulations on Solvated Biomolecular Systems: The Particle Mesh Ewald Method Leads to Stable Trajectories of DNA, RNA, and Proteins. J Am Chem Soc 117: 4193–4194.

[39] Darden T, York D, Pedersen L (1993) Particle mesh Ewald: An N · log(N) method for Ewald sums in large systems. J Chem Phys 98: 10089–10092.

[40] Ryckaert JP, Ciccotti G, Berendsen HJ (1977) Numerical integration of the cartesian equations of motion of a system with constraints: molecular dynamics of n-alkanes. J Comput Phys 23: 327–341.

[41] Petrek M, Kosinov P, Koca J, Otyepka M (2007) MOLE: a Voronoi diagram-based explorer of molecular channels, pores, and tunnels. Structure 15: 1357–1363. 17997961 10.1016/j.str.2007.10.007

[42] Kabsch W, Sander C (1983) Dictionary of protein secondary structure: pattern recognition of hydrogen-bonded and geometrical features. Biopolymers 22: 2577–2637. 6667333 10.1002/bip.360221211

[43] Joosten RP, te Beek TAH, Krieger E, Hekkelman ML, Hooft RWW, et al (2011) A series of PDB related databases for everyday needs. Nucleic Acids Res 39: D411–D419. 21071423 10.1093/nar/gkq1105 PMC3013697

[44] Lawrence MC, Colman PM (1993) Shape complementarity at protein/protein interfaces. J Mol Biol 234: 946–950. 8263940 10.1006/jmbi.1993.1648

[45] Winn MD, Ballard CC, Cowtan KD, Dodson EJ, Emsley P, et al (2011) Overview of the *CCP*4 suite and current developments. Acta Crystallogr Sect D 67: 235–242. 21460441 10.1107/S0907444910045749 PMC3069738

[46] Kollman PA, Massova I, Reyes C, Kuhn B, Huo S, et al (2000) Calculating structures and free energies of complex molecules: combining molecular mechanics and continuum models. Accounts Chem Res 33: 889–897. 10.1021/ar000033j 11123888

[47] Onufriev A, Bashford D, Case DA (2004) Exploring protein native states and large-scale conformational changes with a modified generalized born model. Proteins 55: 383–394. 15048829 10.1002/prot.20033

[48] Humphrey W, Dalke A, Schulten K (1996) VMD: Visual molecular dynamics. J Mol Graphics 14: 33–38. 10.1016/0263-7855(96)00018-5 8744570

[49] Petkova AT, Leapman RD, Guo Z, Yau WM, Mattson MP, et al (2005) Self-propagating, molecular-level polymorphism in Alzheimer’s beta-amyloid fibrils. Science 307: 262–265. 15653506 10.1126/science.1105850

[50] Mahoney MW, Jorgensen WL (2001) Diffusion constant of the TIP5P model of liquid water. J Chem Phys 114: 363–366.

[51] Habicht G, Haupt C, Friedrich RP, Hortschansky P, Sachse C, et al (2007) Directed selection of a conformational antibody domain that prevents mature amyloid fibril formation by stabilizing Abeta protofibrils. Proc Natl Acad Sci U S A 104: 19232–19237. 18042730 10.1073/pnas.0703793104 PMC2148273

[52] Cerf E, Sarroukh R, Tamamizu-Kato S, Breydo L, Derclaye S, et al (2009) Antiparallel beta-sheet: a signature structure of the oligomeric amyloid beta-peptide. Biochem J 421: 415–423. 19435461 10.1042/BJ20090379

[53] Ahmed M, Davis J, Aucoin D, Sato T, Ahuja S, et al (2010) Structural conversion of neurotoxic amyloid-beta(1–42) oligomers to fibrils. Nat Struct Mol Biol 17: 561–567. 20383142 10.1038/nsmb.1799 PMC2922021

[54] Scheidt HA, Morgado I, Rothemund S, Huster D, Fändrich M (2011) Solid-state NMR spectroscopic investigation of A*β* protofibrils: implication of a *β*-sheet remodeling upon maturation into terminal amyloid fibrils. Angew Chem Int Ed Engl 50: 2837–2840. 21387500 10.1002/anie.201007265

[55] Tarus B, Straub JE, Thirumalai D (2006) Dynamics of Asp23-Lys28 salt-bridge formation in Abeta10–35 monomers. J Am Chem Soc 128: 16159–16168. 17165769 10.1021/ja064872y

[56] Chothia C (1973) Conformation of twisted *β*-pleated sheets in proteins. J Mol Biol 75: 295–302. 4728692 10.1016/0022-2836(73)90022-3

[57] Aileen Funke S, van Groen T, Kadish I, Bartnik D, Nagel-Steger L, et al (2010) Oral Treatment with the d-Enantiomeric Peptide D3 Improves the Pathology and Behavior of Alzheimers Disease Transgenic Mice. ACS Chem Neurosci 1: 639–648. 22778851 10.1021/cn100057j PMC3368690

[58] Lakdawala AS, Morgan DM, Liotta DC, Lynn DG, Snyder JP (2002) Dynamics and fluidity of amyloid fibrils: a model of fibrous protein aggregates. J Am Chem Soc 124: 15150–15151. 12487571 10.1021/ja0273290

[59] Wu JW, Breydo L, Isas JM, Lee J, Kuznetsov YG, et al (2010) Fibrillar oligomers nucleate the oligomerization of monomeric amyloid beta but do not seed fibril formation. J Biol Chem 285: 6071–6079. 20018889 10.1074/jbc.M109.069542 PMC2825401

[60] Ma B, Nussinov R (2010) Polymorphic C-terminal beta-sheet interactions determine the formation of fibril or amyloid beta-derived diffusible ligand-like globulomer for the Alzheimer Abeta42 dodecamer. J Biol Chem 285: 37102–37110. 20847046 10.1074/jbc.M110.133488 PMC2978638

[61] Bernstein SL, Dupuis NF, Lazo ND, Wyttenbach T, Condron MM, et al (2009) Amyloid-*β* protein oligomerization and the importance of tetramers and dodecamers in the aetiology of Alzheimer’s disease. Nat Chem 1: 326–331. 20703363 10.1038/nchem.247 PMC2918915

[62] Larson ME, Lesné SE (2012) Soluble A*β* oligomer production and toxicity. J Neurochem 120: 125–139. 10.1111/j.1471-4159.2011.07478.x PMC3254782 22121920

[63] Lesné S, Koh MT, Kotilinek L, Kayed R, Glabe CG, et al (2006) A specific amyloid-*β* protein assembly in the brain impairs memory. Nature 440: 352–357. 16541076 10.1038/nature04533

[64] Lublin AL, Gandy S (2010) Amyloid-beta oligomers: possible roles as key neurotoxins in Alzheimer’s Disease. Mt Sinai J Med 77: 43–49. 20101723 10.1002/msj.20160 PMC3306842

[65] Li MS, Klimov DK, Straub JE, Thirumalai D (2008) Probing the mechanisms of fibril formation using lattice models. J Chem Phys 129: 175101. 19045373 10.1063/1.2989981 PMC2671665

[66] Srivastava A, Balaji PV (2012) Size, orientation and organization of oligomers that nucleate amyloid fibrils: Clues from MD simulations of pre-formed aggregates. Biochim Biophys Acta 1824: 963–973. 22609417 10.1016/j.bbapap.2012.05.003

[67] Zheng J, Ma B, Nussinov R (2006) Consensus features in amyloid fibrils: sheet-sheet recognition via a (polar or nonpolar) zipper structure. Phys Biol 3: P1–P4. 17021379 10.1088/1478-3975/3/3/P01

[68] Dodart JC, Bales KR, Gannon KS, Greene SJ, DeMattos RB, et al (2002) Immunization reverses memory deficits without reducing brain A*β* burden in Alzheimer’s disease model. Nat Neurosci 5: 452–457. 11941374 10.1038/nn842

[69] Hillen H, Barghorn S, Striebinger A, Labkovsky B, Müller R, et al (2010) Generation and therapeutic efficacy of highly oligomer-specific beta-amyloid antibodies. J Neurosci 30: 10369–10379. 20685980 10.1523/JNEUROSCI.5721-09.2010 PMC6634649

[70] Fukumoto H, Tokuda T, Kasai T, Ishigami N, Hidaka H, et al (2010) High-molecular-weight beta-amyloid oligomers are elevated in cerebrospinal fluid of Alzheimer patients. FASEB J 24: 2716–2726. 20339023 10.1096/fj.09-150359

[71] Mantyh PW, Ghilardi JR, Rogers S, DeMaster E, Allen CJ, et al (1993) Aluminum, iron, and zinc ions promote aggregation of physiological concentrations of beta-amyloid peptide. J Neurochem 61: 1171–1174. 8360682 10.1111/j.1471-4159.1993.tb03639.x

[72] Klement K, Wieligmann K, Meinhardt J, Hortschansky P, Richter W, et al (2007) Effect of different salt ions on the propensity of aggregation and on the structure of Alzheimer’s abeta(1–40) amyloid fibrils. J Mol Biol 373: 1321–1333. 17905305 10.1016/j.jmb.2007.08.068

[73] Barrow CJ, Zagorski MG (1991) Solution structures of beta peptide and its constituent fragments: relation to amyloid deposition. Science 253: 179–182. 1853202 10.1126/science.1853202

[74] Fraser PE, Nguyen JT, Surewicz WK, Kirschner DA (1991) pH-dependent structural transitions of Alzheimer amyloid peptides. Biophys J 60: 1190–1201. 1760507 10.1016/S0006-3495(91)82154-3 PMC1260174

