# Supplementary material for: Conformational Stability of Fibrillar Amyloid-Beta Oligomers via Protofilament Pair Formation – A Systematic Computational Study
Source: PLoS One. 2013 Jul 31;8(7):e70521. doi: 10.1371/journal.pone.0070521 (PMC3729696; doi:10.1371/journal.pone.0070521)
Supplement: File S1 — Figures S1–S22 & Table S1. (PDF) [file pone.0070521.s001.pdf]

Supporting Information

Conformational Stability of Fibrillar Amyloid-beta Oligomers via Protofilament Pair Formation – A Systematic Computational Study

Anna Kahler[1], Heinrich Sticht[1], Anselm H.C. Horn[1,*]

1 Bioinformatik, Institut für Biochemie, Friedrich-Alexander-Universität Erlangen-Nürnberg, Erlangen, Germany

∗ E-mail: anselm.horn@biochem.uni-erlangen.de

List of Figures

S1 Small protofilaments O_4, O_5, and O_6 with their initial structure and the structure after 50 ns of simulation. 3
S2 Medium-sized protofilaments O_8, O_{10}, and O_{12} with their initial structure and the structure after 50 ns of simulation. 4
S3 Large protofilaments O_{24} and O_{48} with their initial structure and the structure after 50 ns of simulation. 5
S4 Tiny protofilament pairs $O_{2\times1}$, $O_{2\times2}$, and $O_{2\times3}$ with their initial structure and the structure after 50 ns of simulation. 6
S5 Small protofilament pairs $O_{2\times4}$, $O_{2\times5}$, and $O_{2\times6}$ with their initial structure and the structure after 50 ns of simulation. 7
S6 Large protofilament pairs $O_{2\times12}$ and $O_{2\times24}$ with their initial structure and the structure after 50 ns of simulation. 8
S7 Rmsd values for the simulations of the tiny oligomers, 4-mer, 5-mer, 6-mer, 8-mers, and 10-mers. 9
S8 Rmsd values for the simulations of the 12-mer, 24-mers, and 48-mers. 10
S9 Number of hydrogen bonds for the simulations of the tiny oligomers, 4-mer, 5-mer, 6-mer, 8-mers, and 10-mers. 11
S10 Number of hydrogen bonds for the simulations of the 12-mers, 24-mers, and 48-mers. 12
S11 Twist angle of the oligomer for the simulations of the 4-mer, the 5-mer, the 6-mer, and the two 8-mers. 13
S12 Twist angle of the oligomer for the simulations of the 10-mers, 12-mers, 24-mers, and 48-mers. 14
S13 Content of parallel β-sheet for the small oligomers O_4, O_5 and O_6. 15
S14 Content of parallel β-sheet for the oligomers O_8 and $O_{2\times4}$. 16
S15 Content of parallel β-sheet for the oligomers O_{10} and $O_{2\times5}$. . . . 17
S16 Content of parallel β-sheet for the oligomers O_{12} and $O_{2\times6}$. . . . 18
S17 Content of parallel β-sheet for the large protofilament O_{24}. . . . 19
S18 Content of parallel β-sheet for the large protofilament pair $O_{2\times12}$. 20
S19 Content of parallel β-sheet for the large protofilament O_{48}. . . . 21
S20 Content of parallel β-sheet for the large protofilament O_{48}. . . . 22
S21 Content of parallel β-sheet for the large protofilament pair $O_{2\times24}$. 23
S22 Content of parallel β-sheet for the large protofilament pair $O_{2\times24}$. 24

List of Tables

S1 Appearance of inter- and intramolecular salt bridges (in %) in the MD simulation. 25

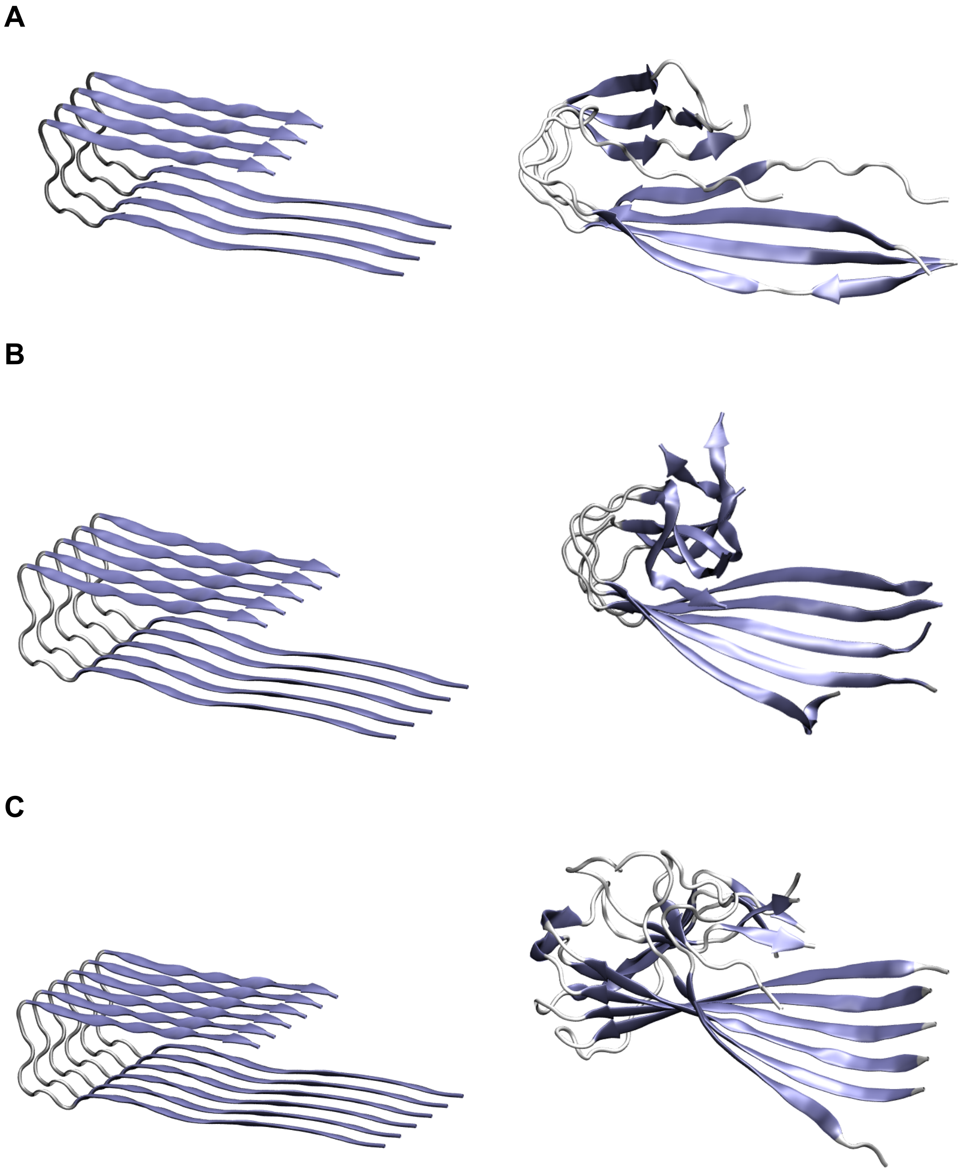

Figure S1. Small protofilaments O_4, O_5, and O_6 with their initial structure and the structure after 50 ns of simulation. The upper C-terminal chains in O_4 (A) bend upwards to cover the hydrophobic residues pointing towards the solvent. This flexible hinge can also observed in the other oligomers O_6 and O_6; in the 5-mer (B) the twisting of parallel β-sheets can already be observed but it is more obvious in the 6-mer (C).

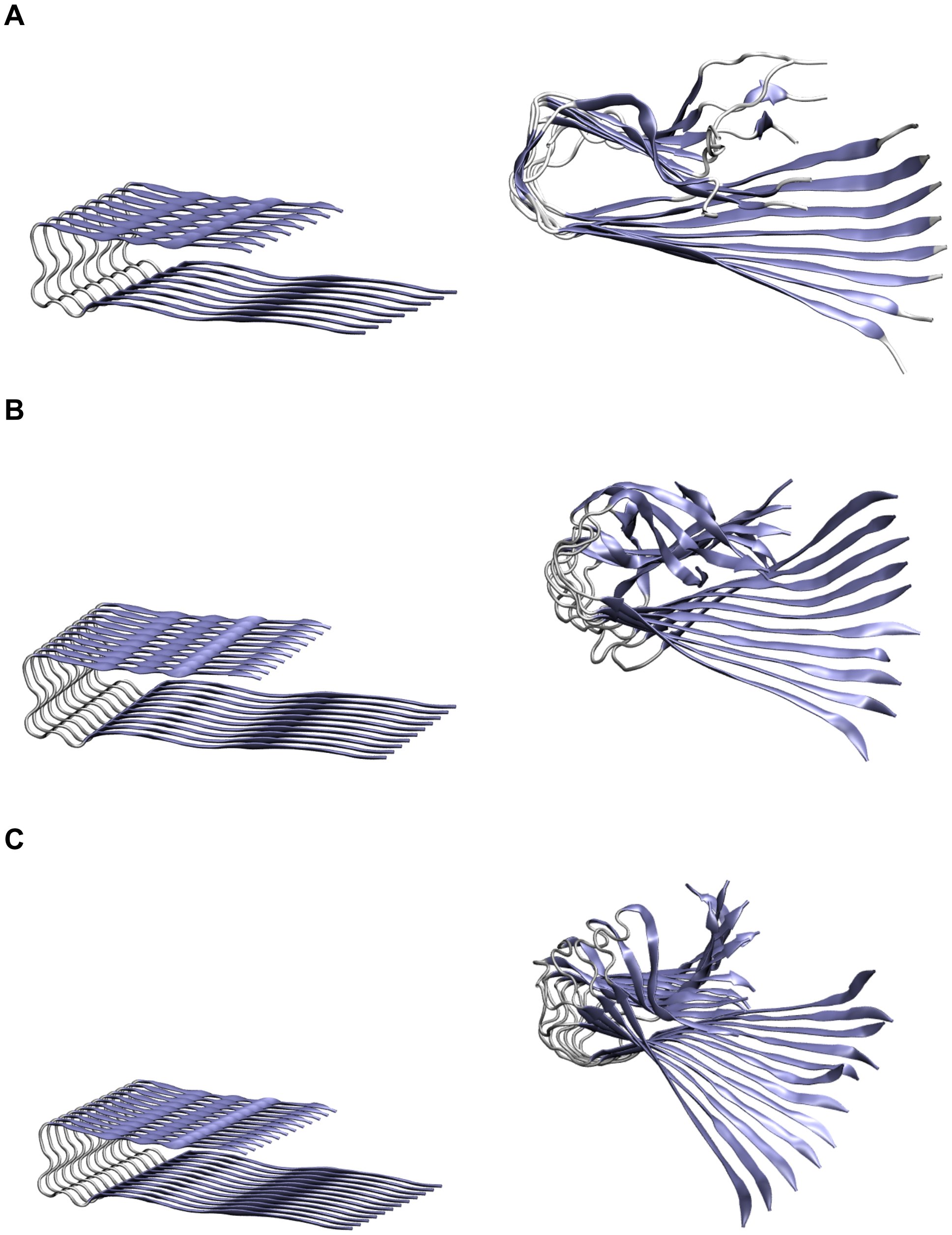

Figure S2. Medium-sized protofilaments O_8, O_{10}, and O_{12} with their initial structure and the structure after 50 ns of simulation. Twisting of parallel β-sheets is strongly pronounced in oligomers with around 10 monomers. In the 8-mer (A) the flexible hinge is present in all C-terminal chains whereas it is less present in the middle chains of the 10-mer (B) and the 12-mer (C).

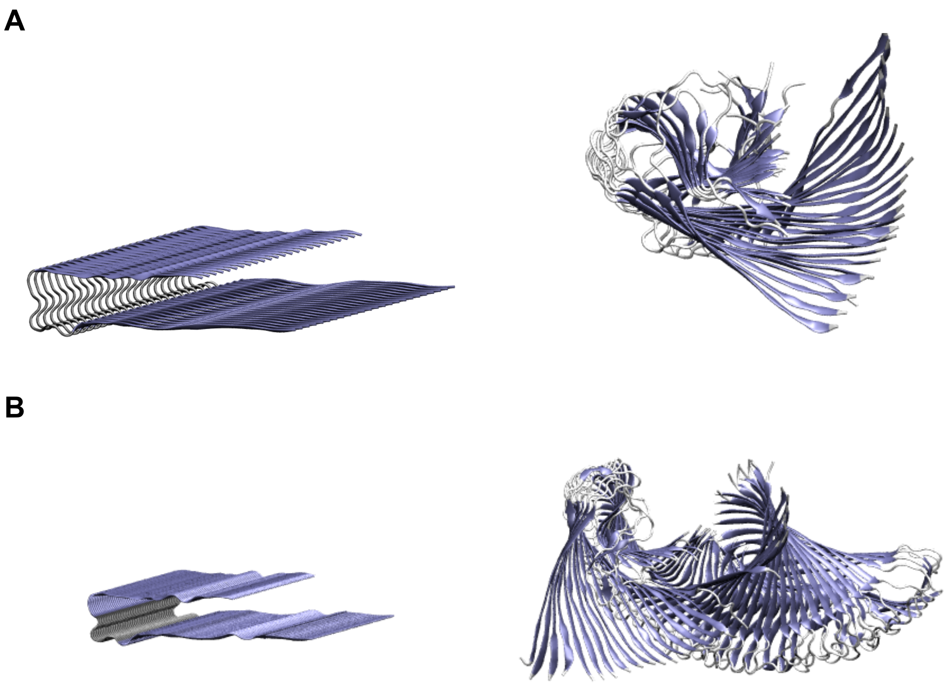

Figure S3. Large protofilaments O_{24} and O_{48} with their initial structure and the structure after 50 ns of simulation. The most obvious structural property in the large oligomers is the extensive twist around the elongation axis. The 24-mer (A) twists about 110° and the 48-mer (B) nearly one way around (335°). The flexible hinge is only present in the C-termini of the outer chains.

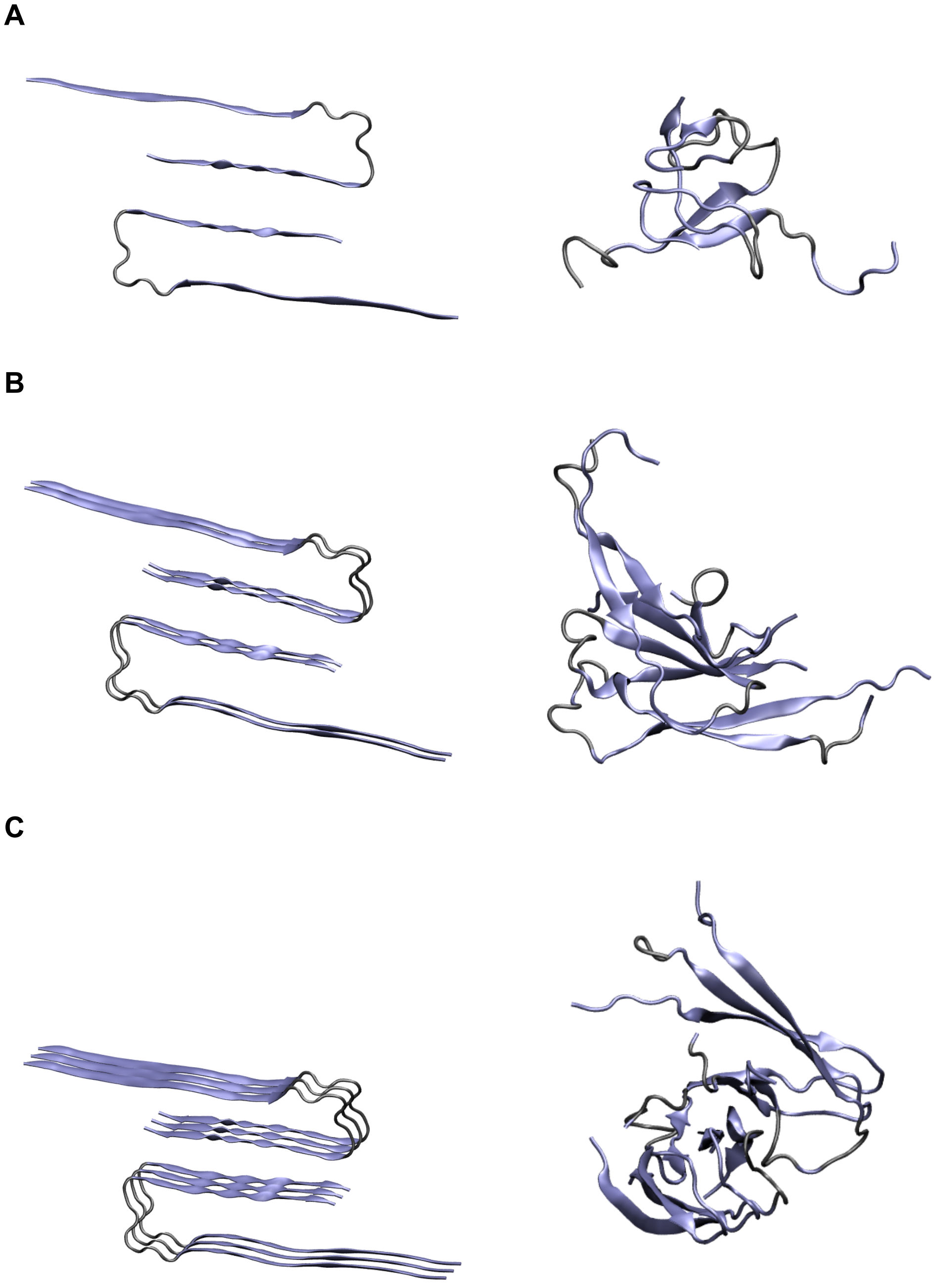

Figure S4. Tiny protofilament pairs $O_{2\times1}$, $O_{2\times2}$, and $O_{2\times3}$ with their initial structure and the structure after 50 ns of simulation. (A) The protofilament pair dimer completely lost its initial conformation. (B) The protofilament pair tetramer rearranges to hide hydrophobic residues from the solvent. (C) The protofilament pair hexamer keeps the conformation of the trimer parts but rearranges to cover hydrophobic residues.

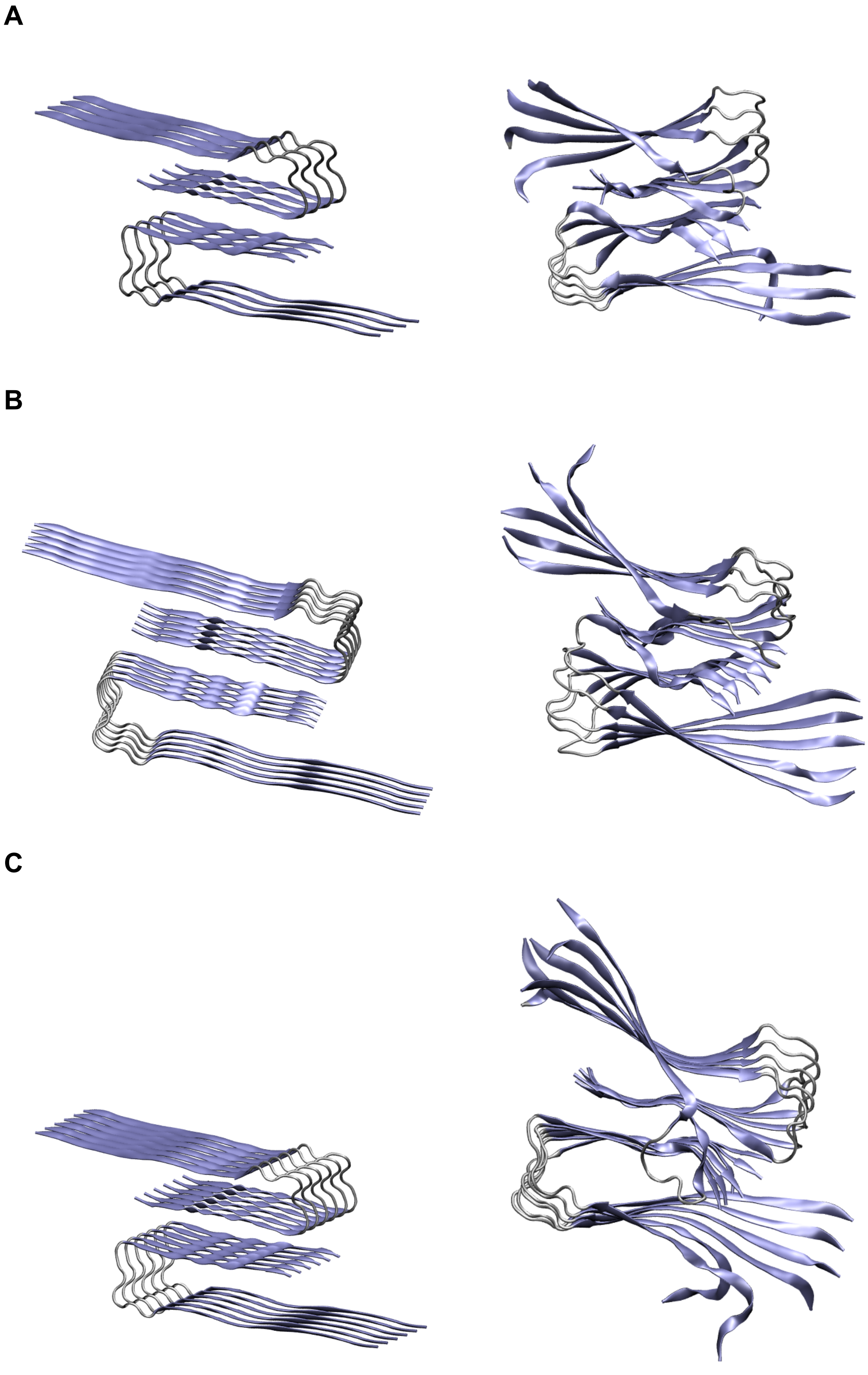

Figure S5. Small protofilament pairs $O_{2\times4}$, $O_{2\times5}$, and $O_{2\times6}$ with their initial structure and the structure after 50 ns of simulation. These oligomers exhibit structural properties of their smaller building blocks: the twist angle and the flexible hinge although the latter is not that much present due to the C-terminal contacts. The 8-mer (A) reveals a larger twist that the 12-mer (C) because the larger hydrophobic interface in the 12-mer compensates the twist of parallel β-sheets. The 10-mer (B) combines properties of the other two: a large twist and a medium hydrophobic interface.

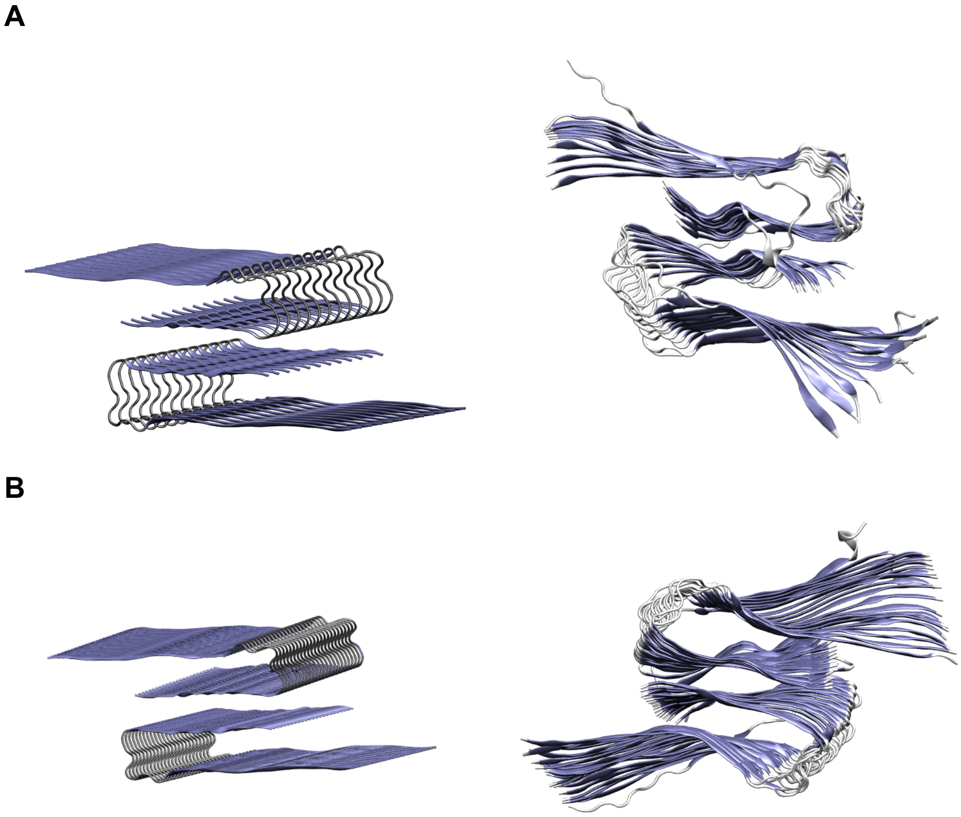

Figure S6. Large protofilament pairs $O_{2\times 12}$ and $O_{2\times 24}$ with their initial structure and the structure after 50 ns of simulation. The hydrophobic interaction via the C-terminal interface diminishes the twisting effect of parallel β-sheets. The 24-mer (A) twists much less than its protofilament counterpart (Figure S3A); the same can be seen for the 48-mer (B) and its protofilament counterpart (Figure S3B).

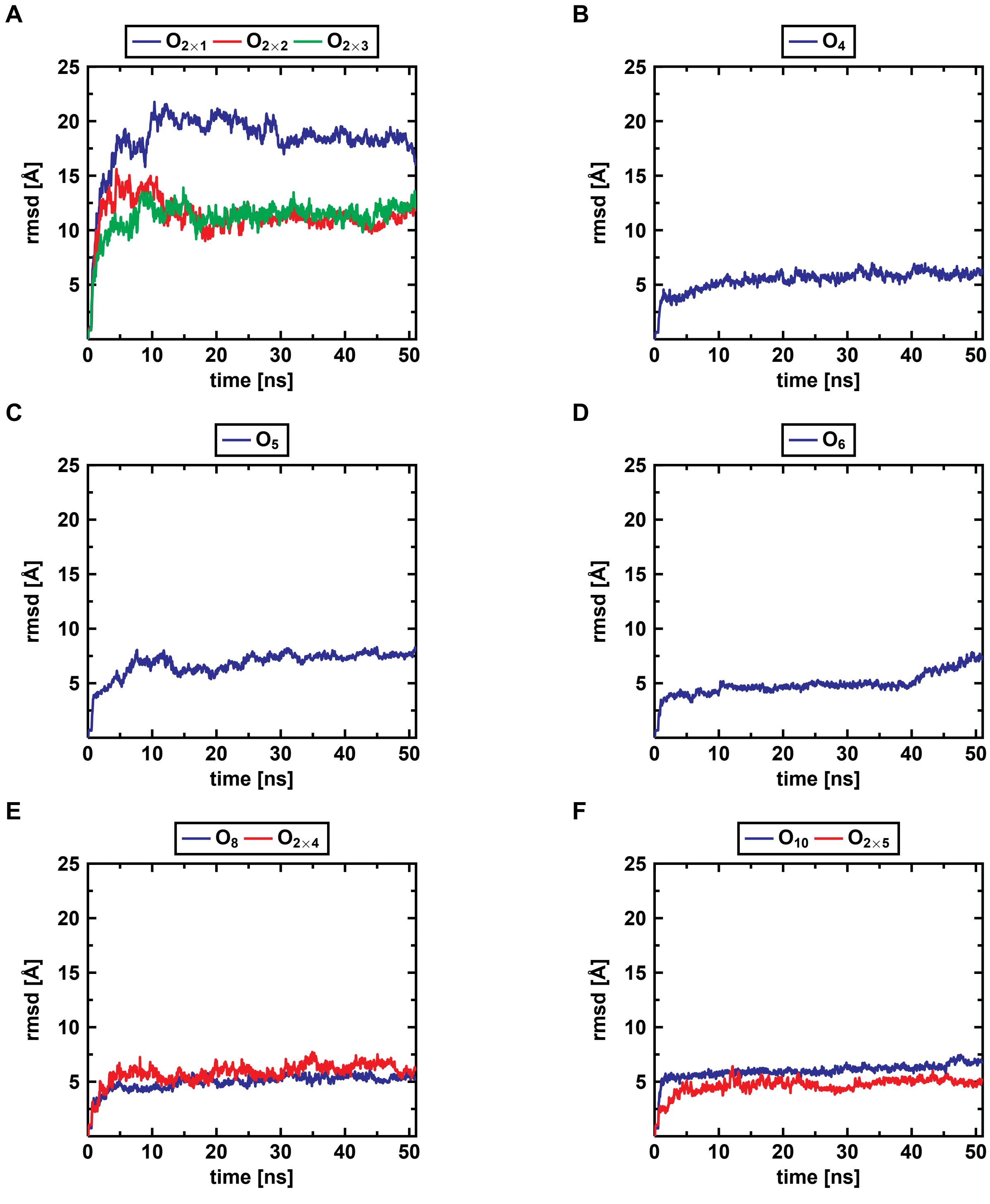

Figure S7. Rmsd values for the simulations of the tiny oligomers, 4-mer, 5-mer, 6-mer, 8-mers, and 10-mers. The values of the tiny oligomers' simulations (A) are too large to describe stable simulations. The values of the other simulations (B–F) are in the range for simulations of globular shaped proteins.

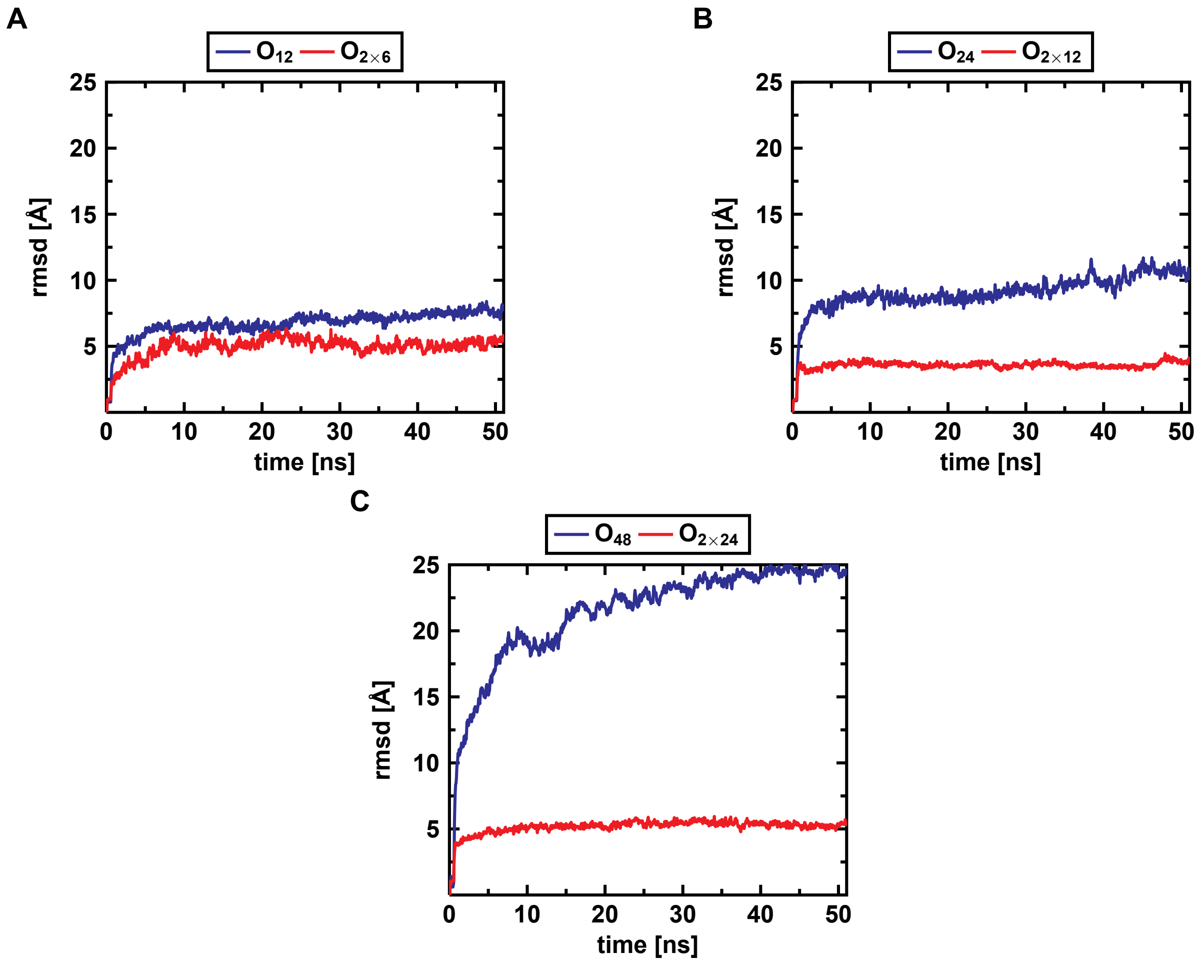

Figure S8. Rmsd values for the simulations of the 12-mer, 24-mers, and 48-mers. The rmsd-values of the protofilament pair simulations (red lines in A–C) are in the range for simulaions of globular shaped proteins and indicate stable simulations. Increasing values of the protofilaments (blue lines in A–C) suggest either unstable simulations or severe distortions in the system.

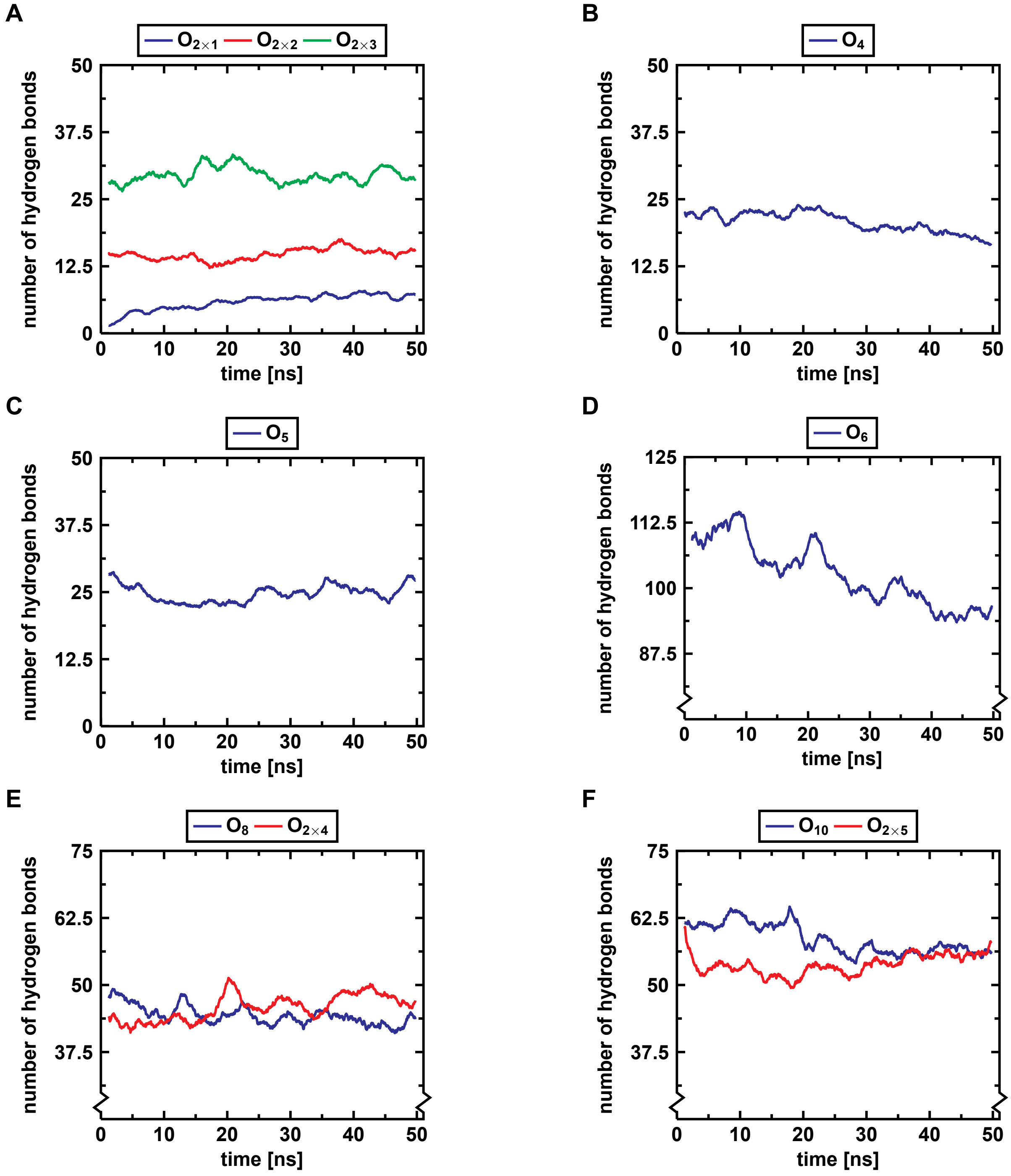

Figure S9. Number of hydrogen bonds for the simulations of the tiny oligomers, 4-mer, 5-mer, 6-mer, 8-mers, and 10-mers. (A) The tiny protofilament pairs $O_{2\times1}$, $O_{2\times2}$, and $O_{2\times3}$. (B) The protofilament tetramer. (C) The protofilament pentamer. (D) The protofilament hexamer. (E) The protofilament and the protofilament pair octamer. (F) The protofilament and the protofilament pair decamer.

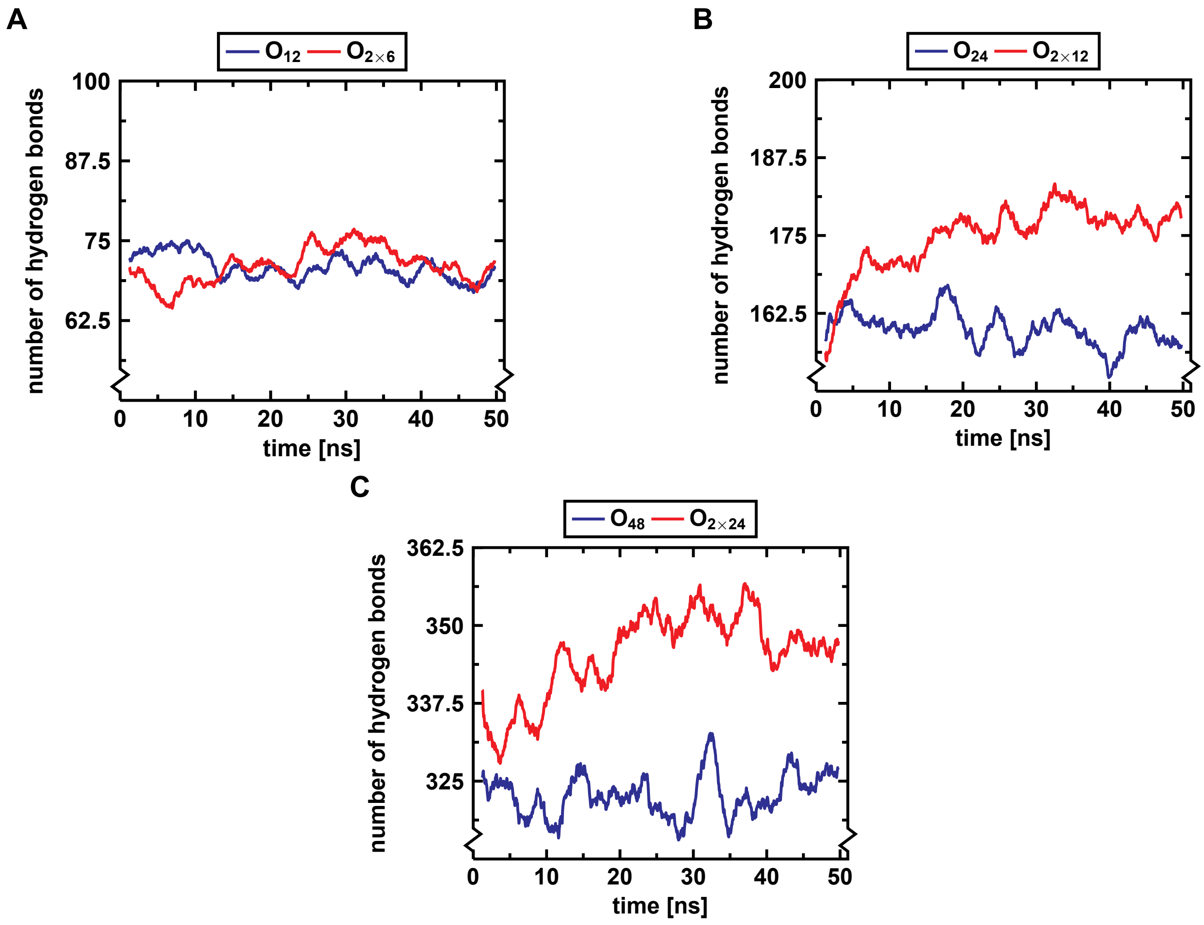

Figure S10. Number of hydrogen bonds for the simulations of the 12-mers, 24-mers, and 48-mers. (A) The protofilament and the protofilament pair dodecamer. (B) The protofilament and the protofilament pair 24-mer. (C) The protofilament and the protofilament pair 48-mer.

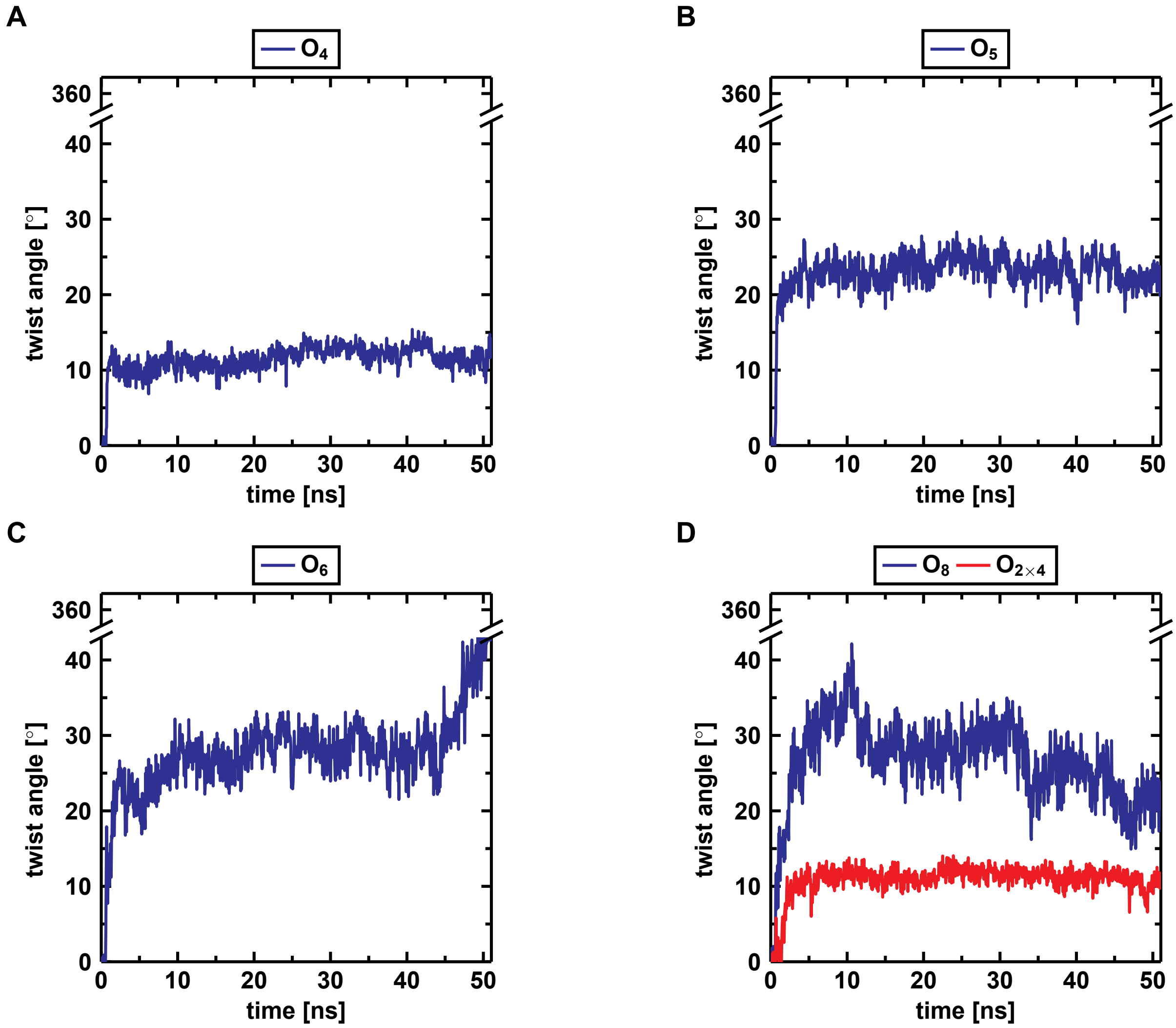

Figure S11. Twist angle of the oligomer for the simulations of the 4-mer, the 5-mer, the 6-mer, and the two 8-mers. (A) The twist angle for the protofilament tetramer. (B) The twist angle for the protofilament pentamer. (C) The twist angle for the protofilament hexamer. (D) The twist angles for the protofilament and the protofilament pair octamers.

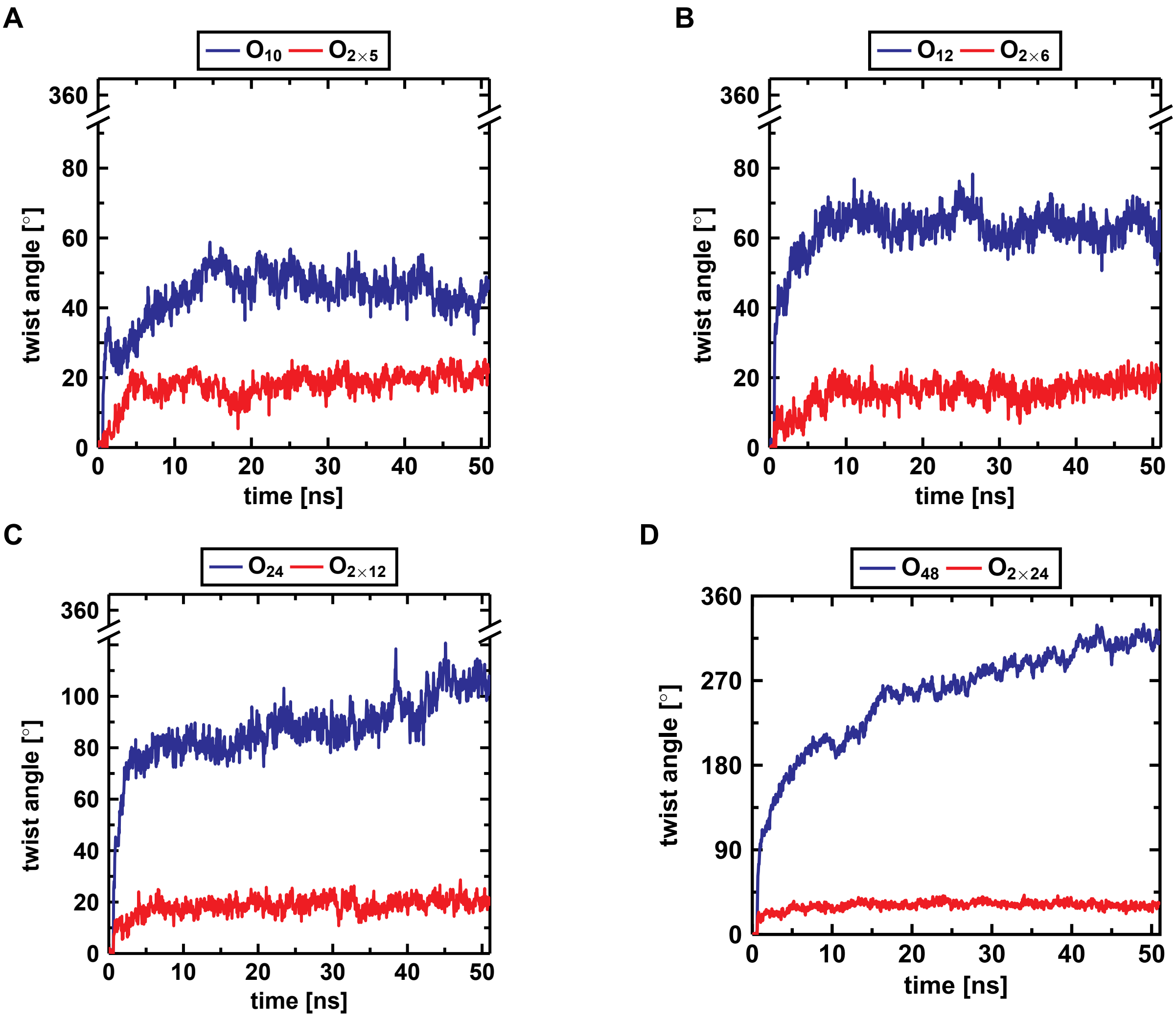

Figure S12. Twist angle of the oligomer for the simulations of the 10-mers, 12-mers, 24-mers, and 48-mers. (A) The twist angles for the protofilament and tha protofilament pair 10-mers. (B) The twist angles for the protofilament and the protofilament pair 12-mers. (C) The twist angles for the protofilament and the protofilament pair 24-mers. (D) The twist angles for the protofilament and the protofilament pair 48-mers.

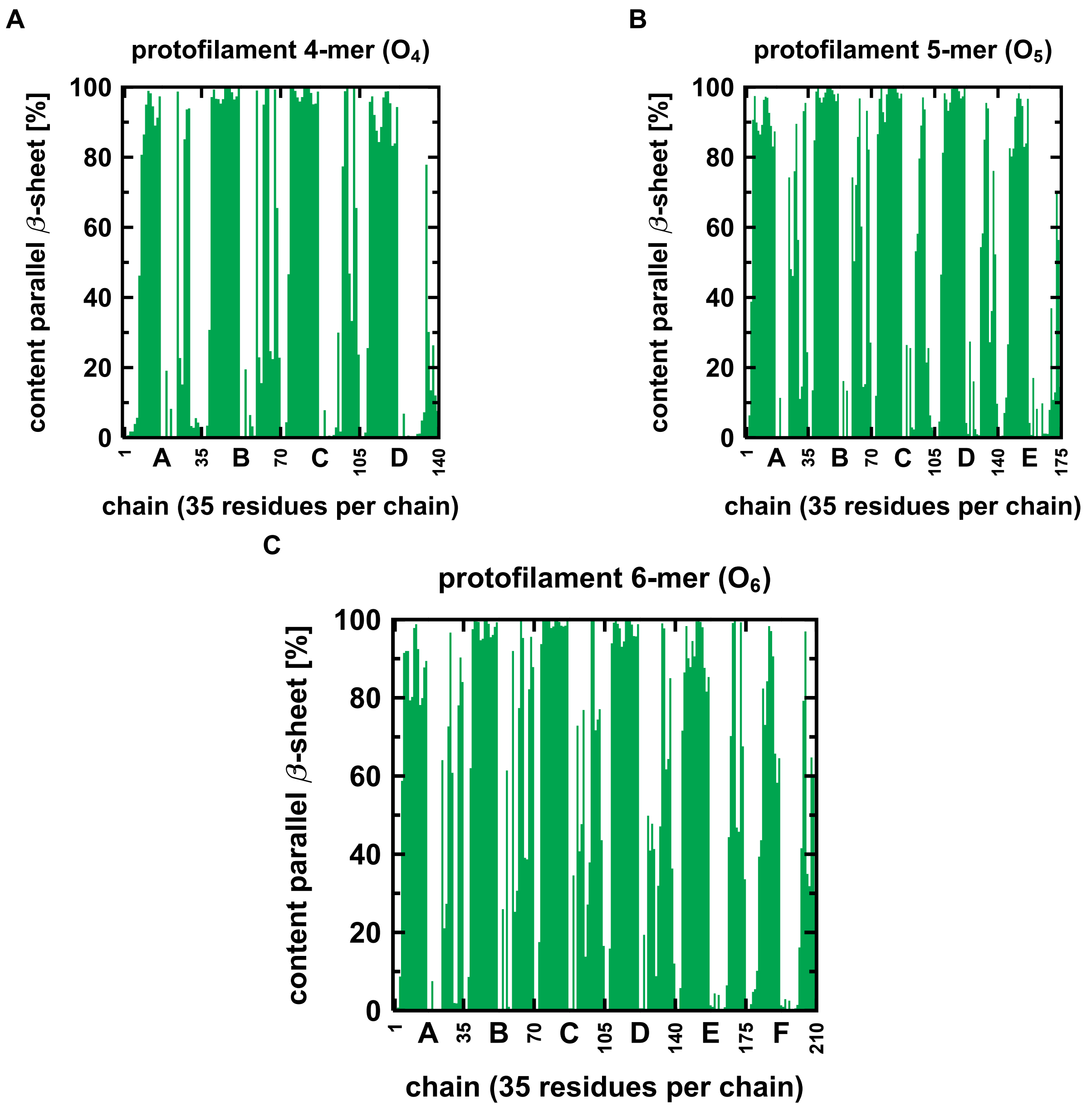

Figure S13. Content of parallel β-sheet for the small oligomers O_4, O_5 and O_6. (A) The protofilament tetramer. (B) The protofilament pentamer. (C) The protofilament hexamer.

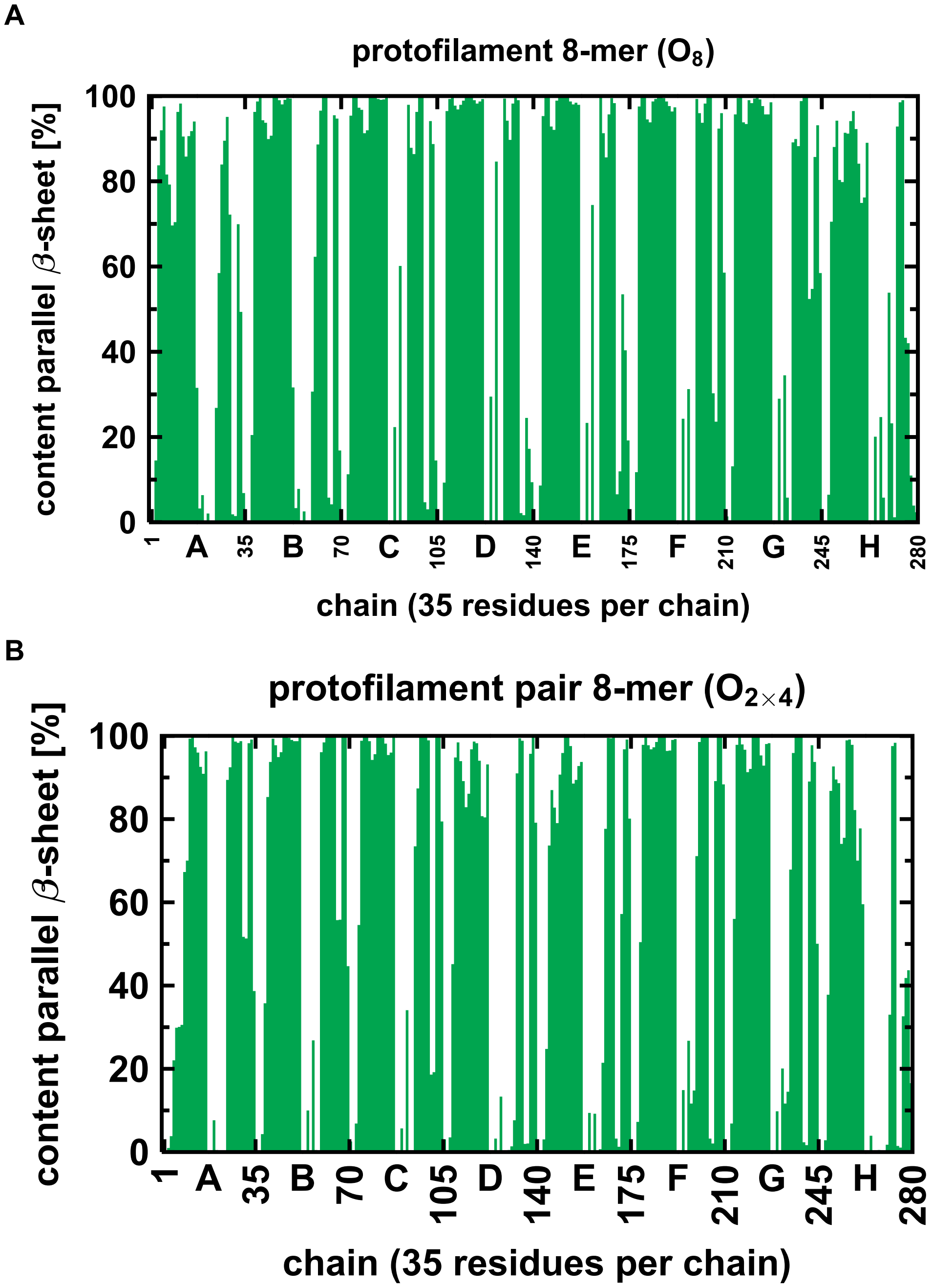

Figure S14. Content of parallel β-sheet for the oligomers O_8 and $O_{2\times4}$. (A) The protofilament octamer. (B) The protofilament pair octamer.

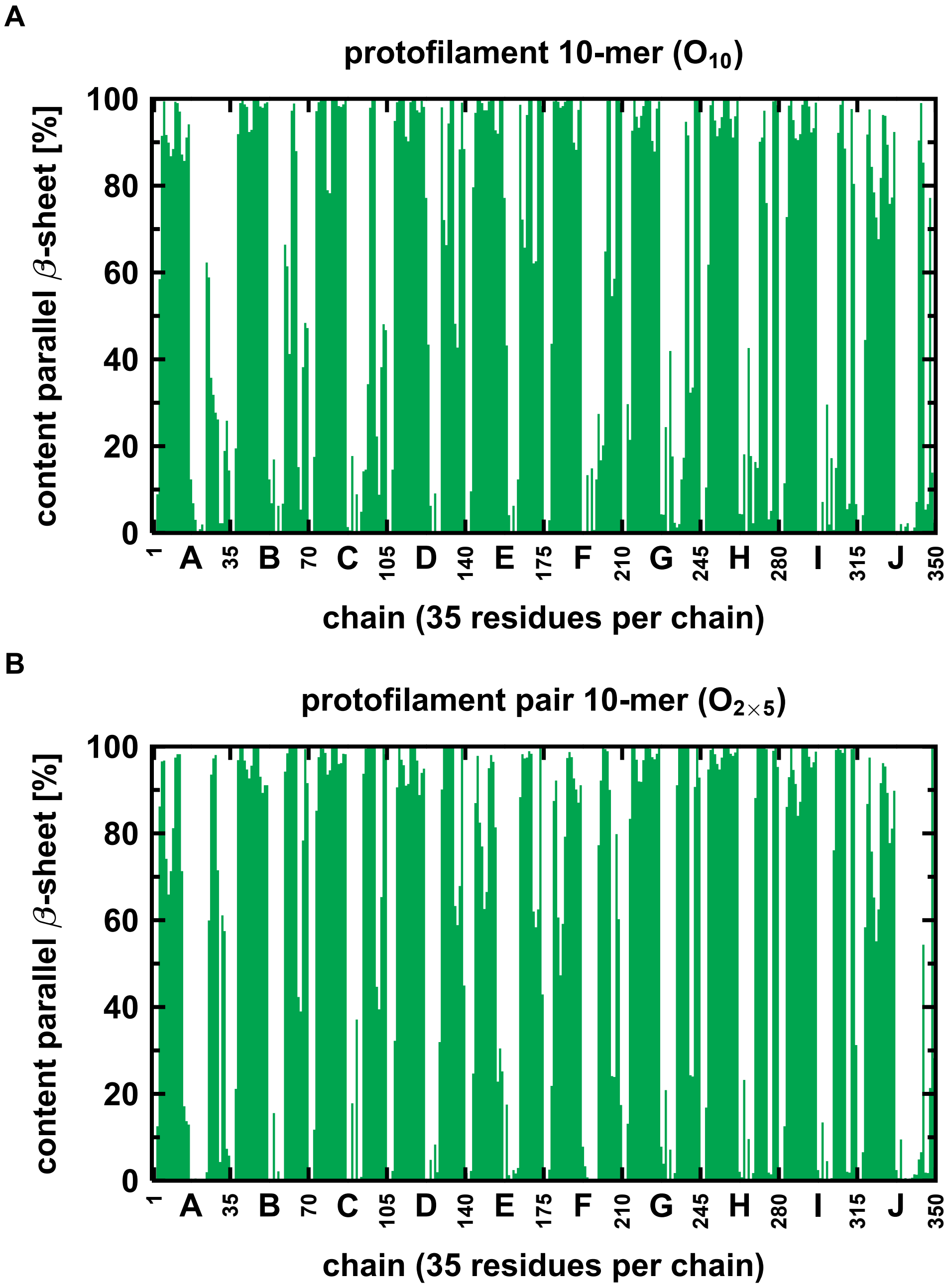

Figure S15. Content of parallel β-sheet for the oligomers O_{10} and $O_{2\times5}$. (A) The protofilament decamer. (B) The protofilament pair decamer.

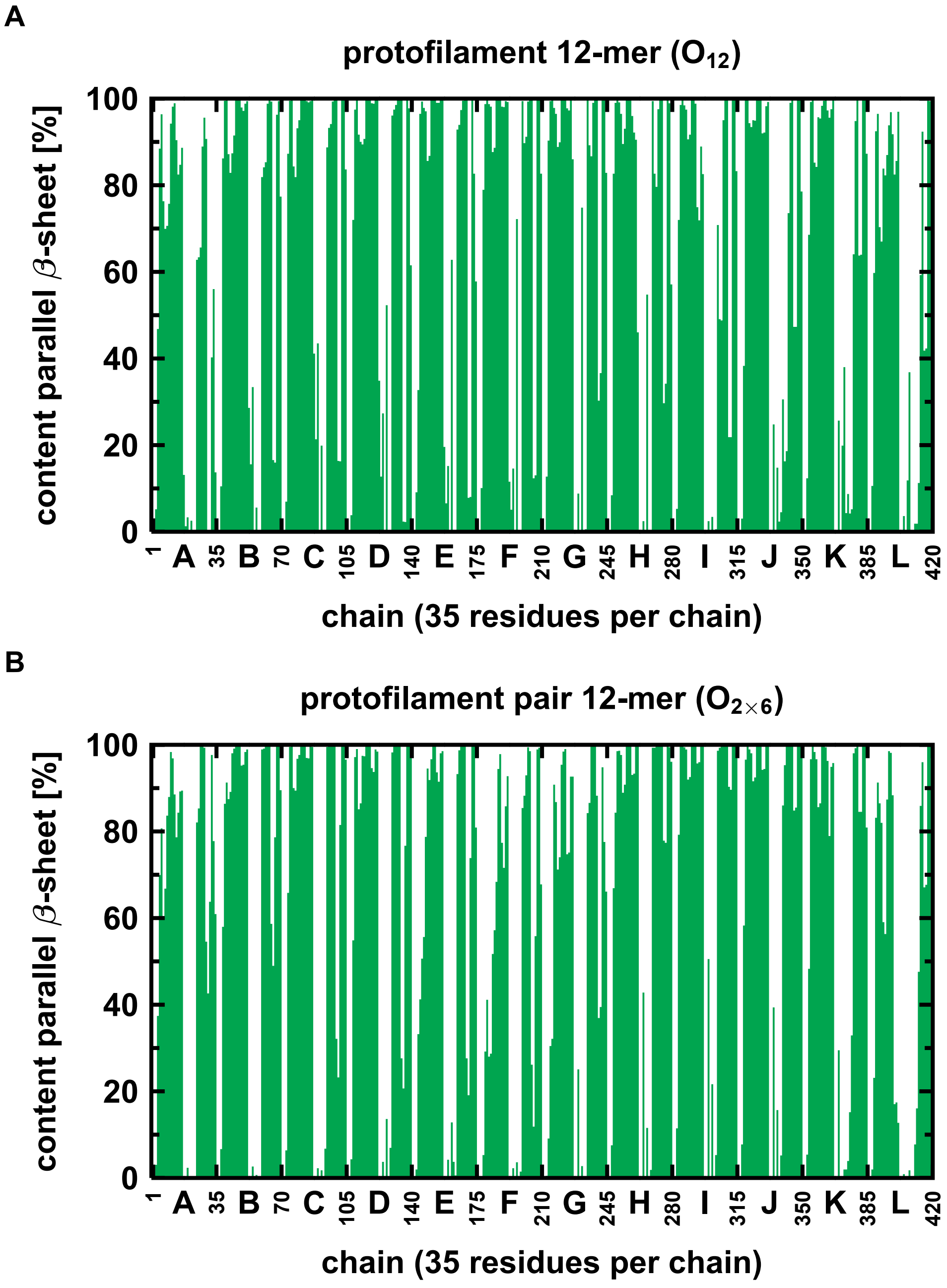

Figure S16. Content of parallel β-sheet for the oligomers O_{12} and $O_{2\times6}$. (A) The protofilament dodecamer. (B) The protofilament pair dodecamer.

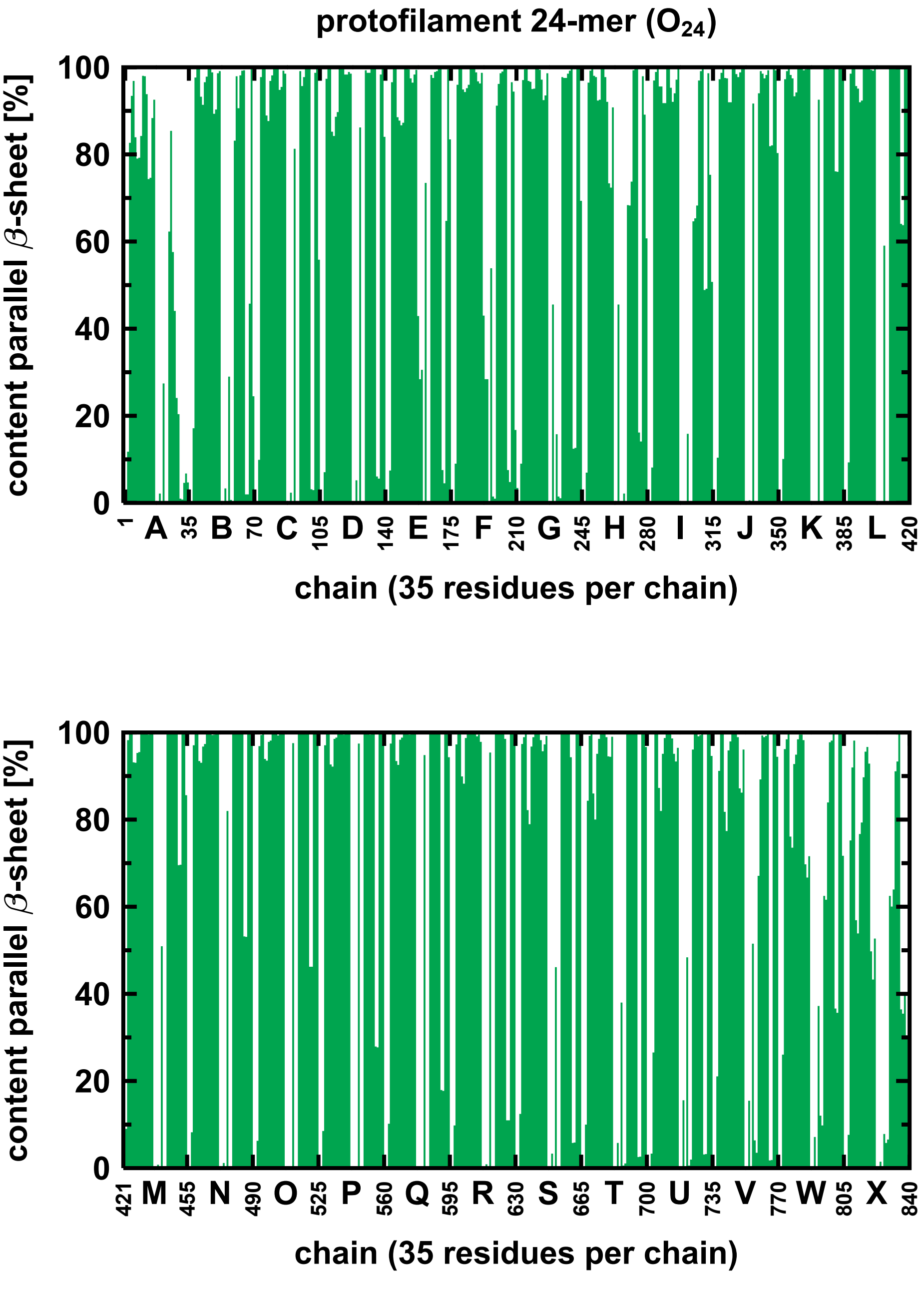

Figure S17. Content of parallel β-sheet for the large protofilament O_{24}.

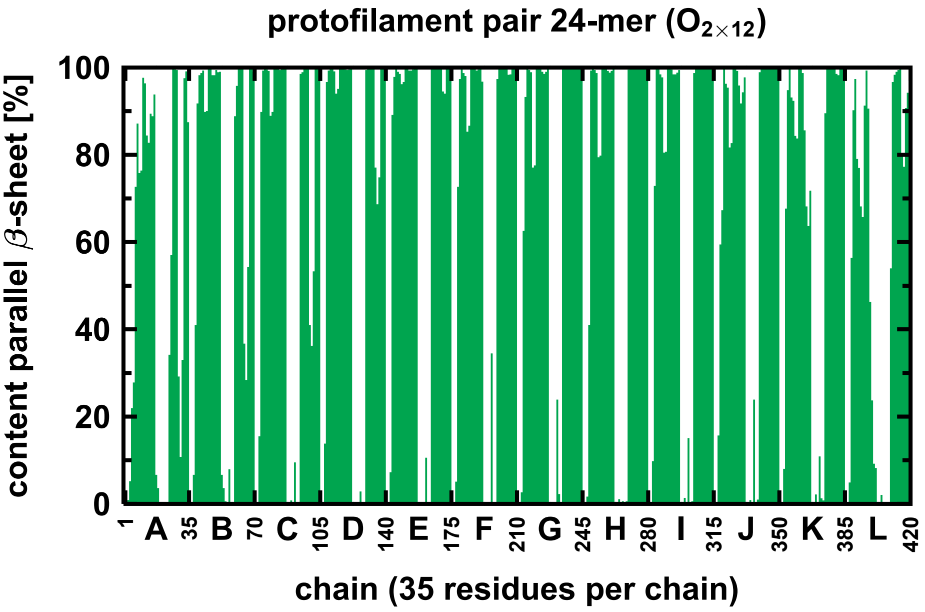

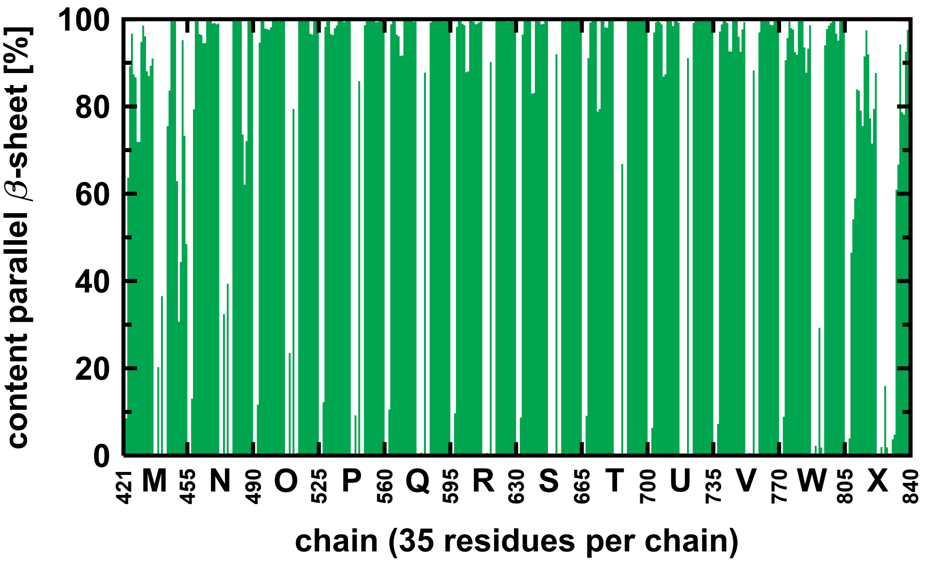

Figure S18. Content of parallel β-sheet for the large protofilament pair $O_{2\times12}$.

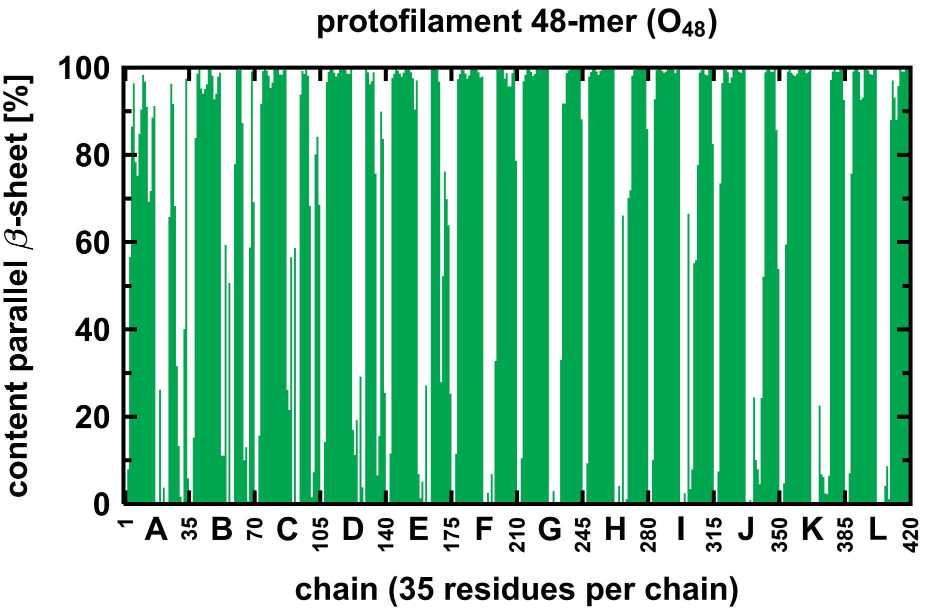

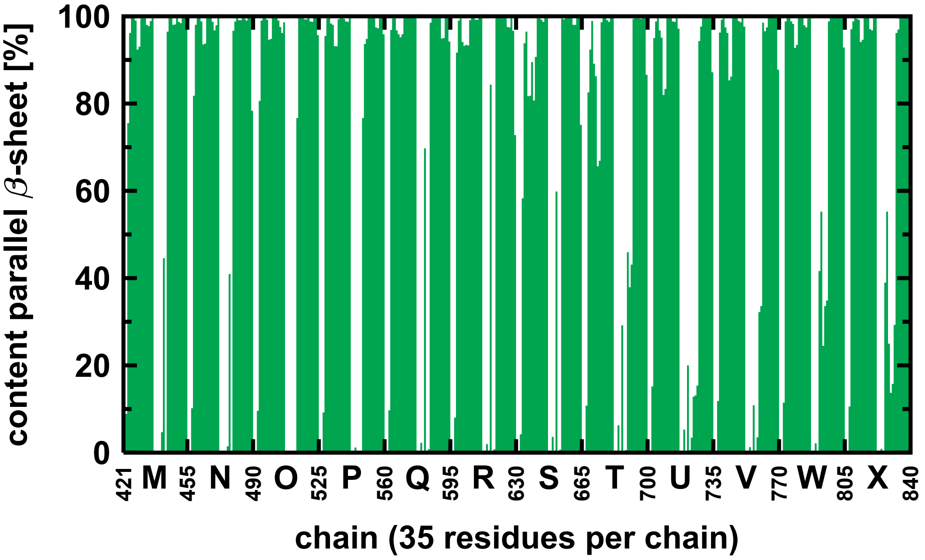

Figure S19. Content of parallel β-sheet for the large protofilament O_{48}. Depicted are the first 24 Aβ chains.

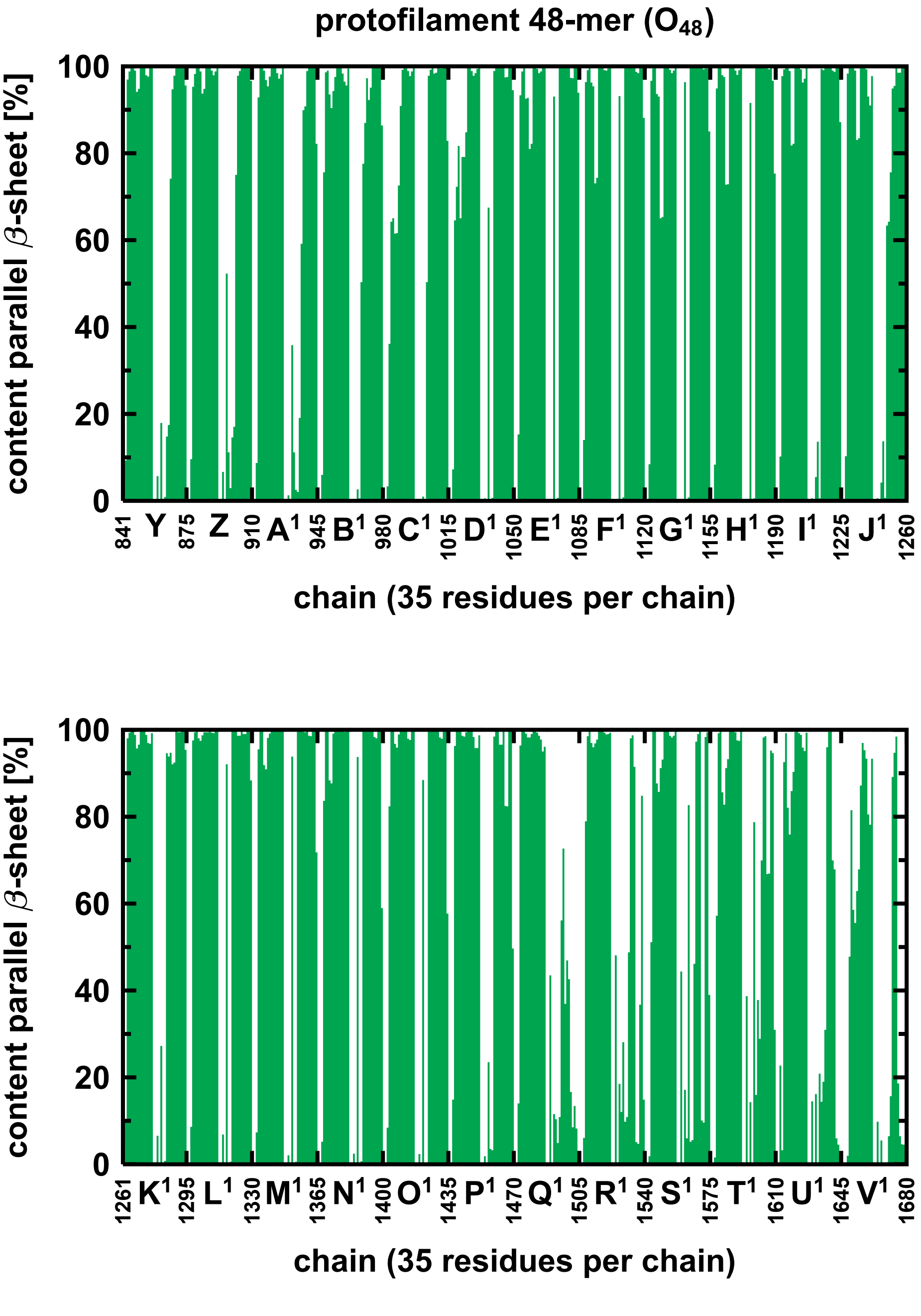

Figure S20. Content of parallel β-sheet for the large protofilament O_{48}. Depicted are the last 24 Aβ chains.

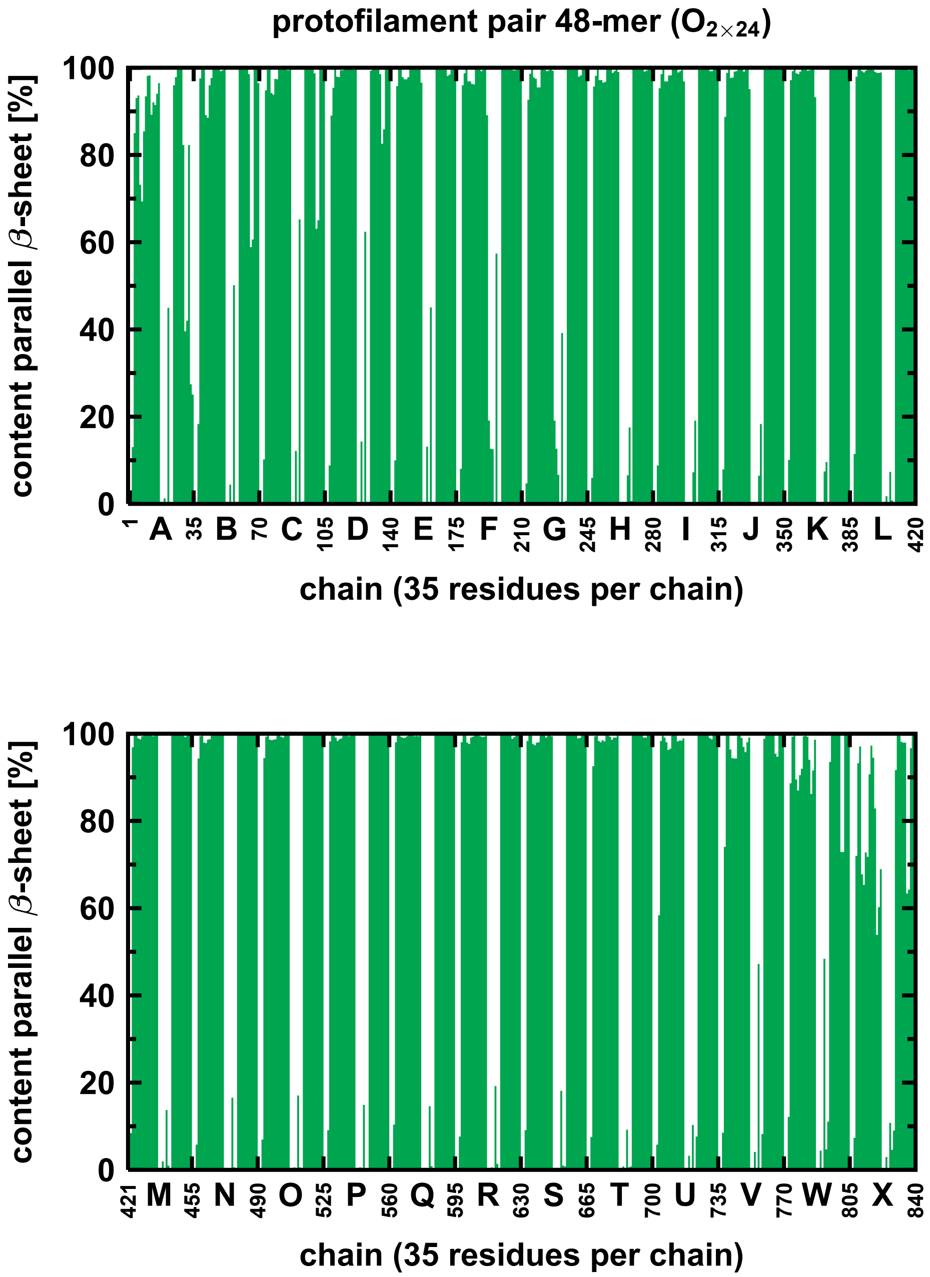

Figure S21. Content of parallel β-sheet for the large protofilament pair $O_{2\times24}$. Depicted are the first 24 Aβ chains.

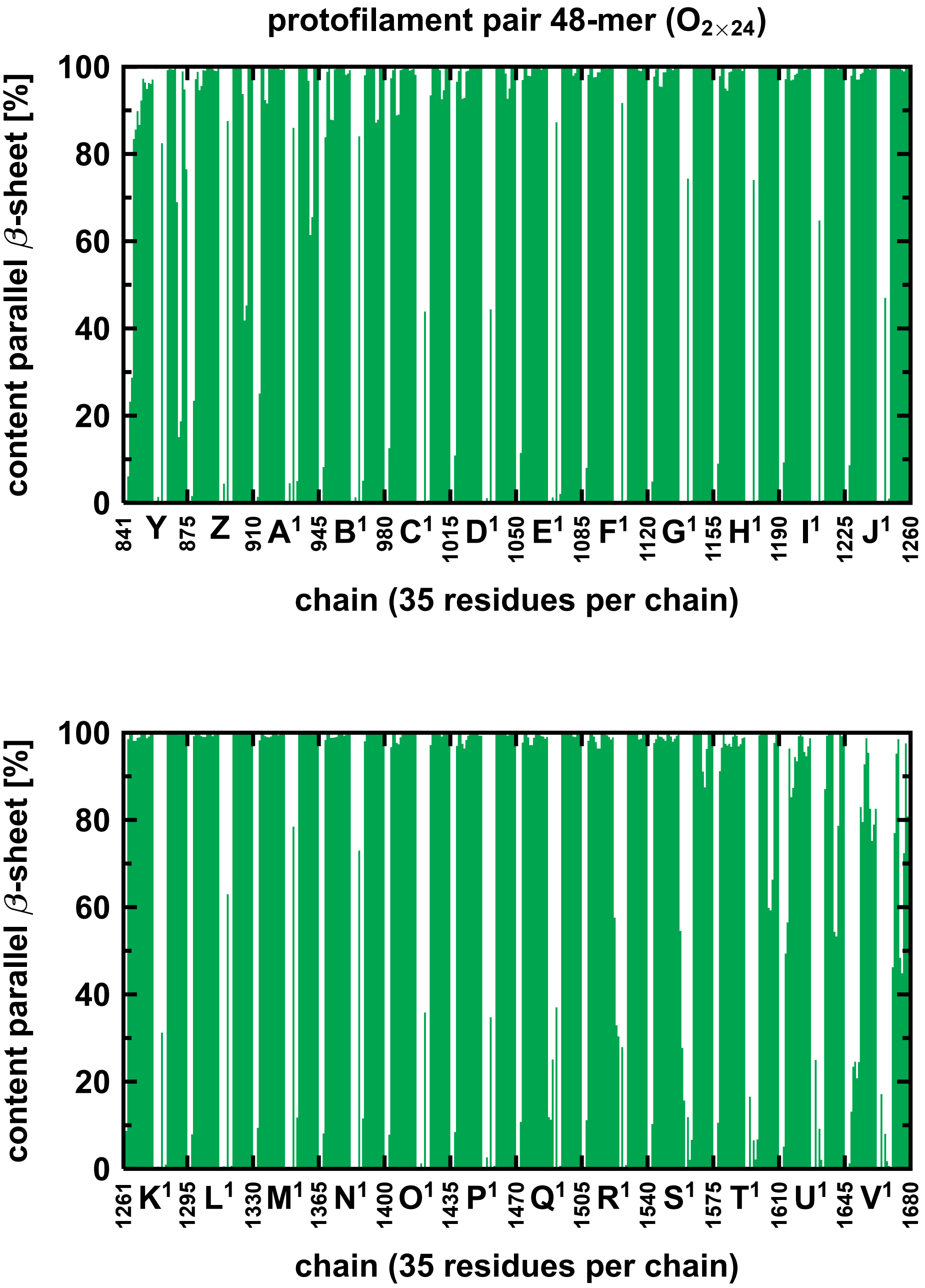

Figure S22. Content of parallel β-sheet for the large oligomer $O_{2\times24}$. Depicted are the last 24 Aβ chains.

Table S1. Appearance of inter- and intramolecular salt bridges (in %) in the MD simulation. Each salt bridge between D23 and K28 has two distances due to the rotation of the carboxylic group of D23; OD1 and OD2 are the two oxygen atoms of the carboxylic group. Electrostatic interaction cutoff distance = 4.2 Å.

	intermolecular			intramolecular				intermolecular			intramolecular		
	**chains**	**OD1**	**OD2**	**chain**	**OD1**	**OD2**		**chains**	**OD1**	**OD2**	**chain**	**OD1**	**OD2**
O_4	**AB**	**81.10**	**83.35**	**A**	**48.38**	**46.23**							
	**BC**	**93.05**	**94.61**	**B**	**91.09**	**59.75**							
	**CD**	**76.79**	**77.08**	**C**	**90.50**	**75.22**							
				**D**	**50.24**	**50.34**							
O_5	**AB**	**92.46**	**90.30**	**A**	**22.53**	**22.53**							
	**BC**	**84.43**	**54.46**	**B**	**89.23**	**86.97**							
	**CD**	**62.00**	**56.81**	**C**	**80.41**	**76.30**							
	**DE**	**21.65**	**19.98**	**D**	**89.32**	**91.67**							
				**E**	**85.70**	**86.29**							
O_6	**AB**	**86.29**	**88.44**	**A**	**27.13**	**24.49**							
	**BC**	**94.91**	**96.87**	**B**	**45.74**	**60.43**							
	**CD**	**87.27**	**87.37**	**C**	**81.39**	**80.41**							
	**DE**	**64.64**	**64.84**	**D**	**90.30**	**84.04**							
	**EF**	**19.78**	**20.67**	**E**	**77.86**	**79.04**							
				**F**	**78.75**	**80.61**							
O_8	**AB**	**81.49**	**74.05**	**A**	**60.43**	**52.40**	$O_{2\times4}$	**AB**	**98.14**	**97.85**	**A**	**42.51**	**47.60**
	**BC**	**99.41**	**98.24**	**B**	**89.62**	**55.04**		**BC**	**95.69**	**96.77**	**B**	**81.00**	**70.71**
	**CD**	**91.38**	**91.28**	**C**	**89.32**	**79.92**		**CD**	**97.85**	**97.16**	**C**	**83.55**	**85.41**
	**DE**	**57.49**	**55.04**	**D**	**83.45**	**92.46**					**D**	**74.53**	**44.66**
	**EF**	**19.20**	**17.04**	**E**	**93.63**	**93.73**		**EF**	**87.95**	**88.74**	**E**	**20.76**	**32.03**
	**FG**	**11.07**	**11.75**	**F**	**96.87**	**94.52**		**FG**	**99.31**	**97.16**	**F**	**77.96**	**56.42**
	**GH**	**13.22**	**13.91**	**G**	**95.10**	**93.93**		**GH**	**44.66**	**44.66**	**G**	**79.04**	**78.55**
				**H**	**90.21**	**90.01**					**H**	**71.99**	**67.09**
O_{10}	**AB**	**84.04**	**86.88**	**A**	**34.87**	**50.05**	$O_{2\times5}$	**AB**	**11.36**	**12.24**	**A**	**13.81**	**16.45**
	**BC**	**84.04**	**84.62**	**B**	**60.14**	**64.25**		**BC**	**94.22**	**93.14**	**B**	**34.48**	**43.58**
	**CD**	**97.26**	**96.57**	**C**	**63.27**	**73.26**		**CD**	**96.67**	**95.10**	**C**	**82.76**	**85.11**
	**DE**	**85.41**	**80.12**	**D**	**78.84**	**83.25**		**DE**	**8.42**	**8.13**	**D**	**76.49**	**79.43**
	**EF**	**39.57**	**100.00**	**E**	**98.82**	**36.83**					**E**	**22.14**	**21.94**
	**FG**	**98.92**	**99.71**	**F**	**43.78**	**82.76**		**FG**	**84.04**	**80.71**	**F**	**13.12**	**16.06**
	**GH**	**98.04**	**98.53**	**G**	**86.78**	**86.39**		**GH**	**96.18**	**97.45**	**G**	**46.82**	**80.31**
	**HI**	**98.73**	**97.55**	**H**	**73.75**	**93.05**		**HI**	**67.78**	**68.36**	**H**	**94.61**	**71.79**
	**IJ**	**48.09**	**47.40**	**I**	**70.91**	**91.09**		**IJ**	**48.48**	**47.89**	**I**	**91.09**	**95.89**
				**J**	**80.90**	**80.71**					**J**	**83.35**	**77.47**
O_{12}	**AB**	**86.78**	**93.44**	**A**	**44.27**	**31.44**	$O_{2\times6}$	**AB**	**92.85**	**90.99**	**A**	**47.80**	**38.59**
	**BC**	**91.19**	**96.18**	**B**	**47.11**	**43.29**		**BC**	**100.00**	**99.22**	**B**	**85.01**	**88.25**
	**CD**	**97.75**	**97.36**	**C**	**77.47**	**71.01**		**CD**	**67.29**	**68.36**	**C**	**90.99**	**90.99**
	**DE**	**98.53**	**96.77**	**D**	**73.95**	**67.78**		**DE**	**59.16**	**57.49**	**D**	**89.03**	**93.63**
	**EF**	**96.28**	**96.87**	**E**	**73.75**	**80.71**		**EF**	**11.46**	**11.66**	**E**	**90.79**	**90.60**
	**FG**	**95.49**	**94.91**	**F**	**70.52**	**76.00**					**F**	**74.44**	**71.11**
	**GH**	**74.63**	**75.32**	**G**	**78.94**	**56.42**		**GH**	**56.42**	**73.26**	**G**	**68.46**	**65.33**
	**HI**	**19.10**	**18.81**	**H**	**67.48**	**73.95**		**HI**	**72.77**	**69.83**	**H**	**63.08**	**70.03**
	**IJ**	**89.13**	**89.91**	**I**	**32.13**	**34.08**		**IJ**	**37.32**	**38.59**	**I**	**92.07**	**92.26**
	**JK**	**96.67**	**97.94**	**J**	**45.84**	**31.64**		**JK**	**26.25**	**27.72**	**J**	**96.47**	**90.11**
	**KL**	**99.12**	**98.82**	**K**	**76.10**	**82.57**		**KL**	**12.24**	**12.14**	**K**	**89.52**	**89.62**
				**L**	**64.25**	**66.11**					**L**	**25.47**	**22.14**
O_{24}	**AB**	**96.28**	**94.71**	**A**	**54.36**	**61.80**	$O_{2\times12}$	**AB**	**83.25**	**80.71**	**A**	**53.97**	**52.01**
	**BC**	**99.02**	**98.53**	**B**	**90.99**	**86.29**		**BC**	**97.36**	**98.73**	**B**	**76.69**	**91.38**
	**CD**	**97.26**	**93.54**	**C**	**85.01**	**86.19**		**CD**	**99.90**	**99.02**	**C**	**90.70**	**90.99**
	**DE**	**87.86**	**89.03**	**D**	**80.80**	**82.76**		**DE**	**99.02**	**98.92**	**D**	**92.85**	**75.02**
	**EF**	**90.50**	**92.56**	**E**	**81.98**	**81.98**		**EF**	**98.92**	**99.12**	**E**	**96.67**	**88.54**
	**FG**	**85.99**	**85.21**	**F**	**79.24**	**73.85**		**FG**	**96.57**	**96.18**	**F**	**90.11**	**90.70**
	**GH**	**63.76**	**61.31**	**G**	**68.17**	**78.45**		**GH**	**99.80**	**99.12**	**G**	**85.60**	**84.13**
	**HI**	**11.85**	**10.58**	**H**	**65.43**	**65.92**		**HI**	**99.71**	**99.80**	**H**	**82.57**	**86.19**
	**IJ**	**96.87**	**95.20**	**I**	**87.86**	**88.54**		**IJ**	**99.41**	**99.80**	**I**	**81.88**	**86.48**
	**JK**	**98.92**	**99.61**	**J**	**87.86**	**77.57**		**JK**	**99.22**	**99.22**	**J**	**60.82**	**81.98**
	**KL**	**99.71**	**99.80**	**K**	**88.25**	**90.79**		**KL**	**3.23**	**3.33**	**K**	**82.57**	**79.04**
	**LM**	**77.18**	**61.61**	**L**	**37.22**	**87.27**					**L**	**14.79**	**14.89**

Continued on Next Page...

Table S1 – Continued

	intermolecular			intramolecular				intermolecular			intramolecular		
	chains	OD1	OD2	chain	OD1	OD2		chains	OD1	OD2	chain	OD1	OD2
	**MN**	**93.24**	**89.62**	**M**	**87.66**	**92.46**		**MN**	**79.53**	**80.90**	**M**	**59.84**	**64.05**
	**NO**	**98.04**	**97.16**	**N**	**93.34**	**92.46**		**NO**	**88.93**	**93.63**	**N**	**88.93**	**85.90**
	**OP**	**96.67**	**95.49**	**O**	**93.63**	**96.18**		**OP**	**94.22**	**96.28**	**O**	**85.41**	**87.66**
	**PQ**	**99.90**	**99.80**	**P**	**91.87**	**91.19**		**PQ**	**99.71**	**99.02**	**P**	**70.32**	**70.62**
	**QR**	**93.24**	**99.71**	**Q**	**86.97**	**96.38**		**QR**	**98.53**	**98.04**	**Q**	**86.19**	**93.14**
	**RS**	**45.15**	**44.96**	**R**	**69.44**	**69.64**		**RS**	**98.73**	**99.41**	**R**	**84.92**	**85.50**
	**ST**	**37.22**	**38.49**	**S**	**92.07**	**94.22**		**ST**	**99.90**	**99.71**	**S**	**74.73**	**73.95**
	**TU**	**15.67**	**15.28**	**T**	**94.91**	**95.79**		**TU**	**99.51**	**99.90**	**T**	**60.43**	**81.49**
	**UV**	**12.54**	**12.44**	**U**	**95.59**	**95.79**		**UV**	**98.14**	**98.24**	**U**	**83.25**	**90.60**
	**VW**	**11.17**	**11.17**	**V**	**97.16**	**95.59**		**VW**	**73.75**	**75.81**	**V**	**75.22**	**67.09**
	**WX**	**9.21**	**9.30**	**W**	**76.20**	**89.72**		**WX**	**78.45**	**78.75**	**W**	**51.91**	**50.73**
				**X**	**25.66**	**27.23**					**X**	**65.52**	**70.03**
O_{48}	**AB**	**96.08**	**93.14**	**A**	**58.08**	**64.25**	$O_{2\times24}$	**AB**	**84.33**	**85.41**	**A**	**51.32**	**51.32**
	**BC**	**90.30**	**68.66**	**B**	**87.27**	**92.26**		**BC**	**99.31**	**99.51**	**B**	**83.15**	**88.74**
	**CD**	**91.48**	**92.36**	**C**	**83.64**	**84.62**		**CD**	**98.73**	**99.22**	**C**	**94.12**	**95.89**
	**DE**	**98.92**	**98.82**	**D**	**68.76**	**78.84**		**DE**	**86.97**	**80.41**	**D**	**78.65**	**79.53**
	**EF**	**88.15**	**82.86**	**E**	**63.47**	**70.23**		**EF**	**96.77**	**96.67**	**E**	**54.95**	**72.58**
	**FG**	**78.45**	**48.19**	**F**	**31.34**	**43.88**		**FG**	**85.99**	**81.88**	**F**	**87.27**	**92.07**
	**GH**	**99.12**	**99.71**	**G**	**85.01**	**59.84**		**GH**	**99.12**	**99.22**	**G**	**63.76**	**60.92**
	**HI**	**99.31**	**98.82**	**H**	**85.11**	**86.09**		**HI**	**99.90**	**99.51**	**H**	**62.10**	**80.90**
	**IJ**	**96.87**	**90.89**	**I**	**71.30**	**84.23**		**IJ**	**100.00**	**99.71**	**I**	**82.47**	**85.11**
	**JK**	**99.31**	**100.00**	**J**	**91.87**	**94.52**		**JK**	**100.00**	**99.90**	**J**	**55.63**	**94.71**
	**KL**	**100.00**	**99.90**	**K**	**69.93**	**88.34**		**KL**	**96.08**	**94.52**	**K**	**75.61**	**74.14**
	**LM**	**99.61**	**97.36**	**L**	**59.45**	**92.26**		**LM**	**98.73**	**98.92**	**L**	**71.50**	**74.44**
	**MN**	**99.61**	**100.00**	**M**	**98.14**	**89.32**		**MN**	**99.80**	**99.71**	**M**	**76.79**	**78.06**
	**NO**	**100.00**	**100.00**	**N**	**85.01**	**93.24**		**NO**	**99.80**	**100.00**	**N**	**80.90**	**82.96**
	**OP**	**34.77**	**42.70**	**O**	**64.64**	**55.14**		**OP**	**99.80**	**99.90**	**O**	**80.80**	**84.33**
	**PQ**	**96.47**	**97.16**	**P**	**90.99**	**82.86**		**PQ**	**100.00**	**100.00**	**P**	**64.54**	**77.08**
	**QR**	**99.90**	**99.51**	**Q**	**93.05**	**92.75**		**QR**	**99.51**	**99.90**	**Q**	**90.01**	**74.63**
	**RS**	**98.82**	**96.77**	**R**	**86.88**	**95.40**		**RS**	**99.31**	**99.41**	**R**	**58.18**	**88.74**
	**ST**	**91.38**	**95.10**	**S**	**85.80**	**94.03**		**ST**	**99.71**	**99.22**	**S**	**69.64**	**93.83**
	**TU**	**98.92**	**99.71**	**T**	**32.71**	**84.62**		**TU**	**70.71**	**69.15**	**T**	**78.75**	**83.35**
	**UV**	**36.14**	**87.66**	**U**	**95.20**	**94.22**		**UV**	**54.06**	**54.75**	**U**	**87.07**	**82.76**
	**VW**	**77.18**	**74.63**	**V**	**73.26**	**75.61**		**VW**	**54.26**	**53.48**	**V**	**90.21**	**87.76**
	**WX**	**100.00**	**99.90**	**W**	**87.07**	**96.77**		**WX**	**8.23**	**9.21**	**W**	**86.19**	**87.37**
	**XY**	**77.18**	**87.76**	**X**	**90.79**	**22.53**					**X**	**67.19**	**66.90**
	**YZ**	**86.19**	**91.48**	**Y**	**76.00**	**57.79**		**YZ**	**92.36**	**53.67**	**Y**	**42.51**	**63.27**
	**Z**$\mathbf{A^1}$	**89.91**	**95.79**	**Z**	**96.87**	**90.70**		**Z**$\mathbf{A^1}$	**99.61**	**99.80**	**Z**	**85.21**	**92.95**
	$\mathbf{A^1B^1}$	**98.73**	**98.73**	$\mathbf{A^1}$	**76.98**	**43.88**		$\mathbf{A^1B^1}$	**99.61**	**99.90**	$\mathbf{A^1}$	**88.25**	**90.99**
	$\mathbf{B^1C^1}$	**45.74**	**48.48**	$\mathbf{B^1}$	**78.06**	**46.72**		$\mathbf{B^1C^1}$	**77.18**	**75.81**	$\mathbf{B^1}$	**79.43**	**76.10**
	$\mathbf{C^1D^1}$	**95.40**	**95.40**	$\mathbf{C^1}$	**90.40**	**90.30**		$\mathbf{C^1D^1}$	**57.49**	**59.26**	$\mathbf{C^1}$	**66.90**	**70.81**
	$\mathbf{D^1E^1}$	**100.00**	**100.00**	$\mathbf{D^1}$	**91.09**	**87.95**		$\mathbf{D^1E^1}$	**97.45**	**97.26**	$\mathbf{D^1}$	**44.27**	**76.30**
	$\mathbf{E^1F^1}$	**99.22**	**99.22**	$\mathbf{E^1}$	**92.65**	**95.98**		$\mathbf{E^1F^1}$	**99.22**	**99.41**	$\mathbf{E^1}$	**84.04**	**90.11**
	$\mathbf{F^1G^1}$	**100.00**	**100.00**	$\mathbf{F^1}$	**90.79**	**88.93**		$\mathbf{F^1G^1}$	**99.90**	**99.71**	$\mathbf{F^1}$	**65.43**	**91.87**
	$\mathbf{G^1H^1}$	**99.90**	**99.61**	$\mathbf{G^1}$	**85.21**	**87.27**		$\mathbf{G^1H^1}$	**99.22**	**99.90**	$\mathbf{G^1}$	**89.72**	**85.90**
	$\mathbf{H^1I^1}$	**75.91**	**81.59**	$\mathbf{H^1}$	**80.80**	**65.82**		$\mathbf{H^1I^1}$	**99.80**	**98.33**	$\mathbf{H^1}$	**76.59**	**83.55**
	$\mathbf{I^1J^1}$	**45.35**	**46.13**	$\mathbf{I^1}$	**94.81**	**88.74**		$\mathbf{I^1J^1}$	**99.51**	**99.31**	$\mathbf{I^1}$	**72.48**	**76.69**
	$\mathbf{J^1K^1}$	**14.10**	**10.48**	$\mathbf{J^1}$	**67.78**	**79.63**		$\mathbf{J^1K^1}$	**99.51**	**98.82**	$\mathbf{J^1}$	**83.64**	**79.24**
	$\mathbf{K^1L^1}$	**63.17**	**75.51**	$\mathbf{K^1}$	**91.28**	**88.15**		$\mathbf{K^1L^1}$	**99.90**	**99.71**	$\mathbf{K^1}$	**86.29**	**85.31**
	$\mathbf{L^1M^1}$	**88.74**	**87.66**	$\mathbf{L^1}$	**80.90**	**84.92**		$\mathbf{L^1M^1}$	**99.90**	**99.90**	$\mathbf{L^1}$	**60.04**	**81.68**
	$\mathbf{M^1N^1}$	**99.02**	**99.41**	$\mathbf{M^1}$	**87.46**	**90.40**		$\mathbf{M^1N^1}$	**99.90**	**99.61**	$\mathbf{M^1}$	**49.07**	**99.02**
	$\mathbf{N^1O^1}$	**99.02**	**99.22**	$\mathbf{N^1}$	**92.85**	**87.66**		$\mathbf{N^1O^1}$	**99.80**	**100.00**	$\mathbf{N^1}$	**85.50**	**71.50**
	$\mathbf{O^1P^1}$	**61.51**	**53.28**	$\mathbf{O^1}$	**50.24**	**82.66**		$\mathbf{O^1P^1}$	**99.90**	**99.80**	$\mathbf{O^1}$	**87.07**	**61.70**
	$\mathbf{P^1Q^1}$	**39.67**	**37.22**	$\mathbf{P^1}$	**89.62**	**91.67**		$\mathbf{P^1Q^1}$	**99.61**	**99.90**	$\mathbf{P^1}$	**82.47**	**87.76**
	$\mathbf{Q^1R^1}$	**54.75**	**62.88**	$\mathbf{Q^1}$	**90.50**	**90.01**		$\mathbf{Q^1R^1}$	**91.87**	**89.91**	$\mathbf{Q^1}$	**64.64**	**70.13**
	$\mathbf{R^1S^1}$	**98.92**	**99.12**	$\mathbf{R^1}$	**57.00**	**54.85**		$\mathbf{R^1S^1}$	**94.61**	**96.38**	$\mathbf{R^1}$	**50.24**	**51.71**
	$\mathbf{S^1T^1}$	**94.61**	**95.10**	$\mathbf{S^1}$	**88.54**	**87.95**		$\mathbf{S^1T^1}$	**58.57**	**55.04**	$\mathbf{S^1}$	**68.36**	**74.44**
	$\mathbf{T^1U^1}$	**56.32**	**55.04**	$\mathbf{T^1}$	**87.56**	**88.93**		$\mathbf{T^1U^1}$	**27.91**	**29.77**	$\mathbf{T^1}$	**84.04**	**77.86**
	$\mathbf{U^1V^1}$	**41.04**	**41.82**	$\mathbf{U^1}$	**92.95**	**90.60**		$\mathbf{U^1V^1}$	**10.19**	**8.81**	$\mathbf{U^1}$	**94.03**	**94.71**
				$\mathbf{V^1}$	**76.30**	**74.73**					$\mathbf{V^1}$	**79.14**	**87.56**
